# The Impact of Mindfulness as a Session‐Introduction on Symptoms and Alliance in Individual Cognitive‐Behavioral Therapy With Children and Adolescents: A Randomized Controlled Trial

**DOI:** 10.1002/jclp.70216

**Published:** 2026-08-29

**Authors:** Julia Kalmar, Ines Baumann, Elena Schleiting, Anne Grünert, Eva Vonderlin, Katajun Lindenberg, Andreas B. Neubauer, Thomas Heidenreich, Johannes Mander

**Affiliations:** ^1^ Center for Psychological Psychotherapy, Institute of Psychology Ruprecht‐Karls‐University of Heidelberg Heidelberg Germany; ^2^ Department of Developmental Psychology Institute of Psychology, Ruprecht‐Karls‐University of Heidelberg Heidelberg Germany; ^3^ Department of Developmental Psychology and Research Methods, Institute of Psychology RWTH Aachen‐University Aachen Germany; ^4^ Department of Education and Human Development Leibniz Institute for Research and Information in Education (DIPF) Frankfurt Germany; ^5^ Department of Clinical Psychology of Childhood and Adolescence, Institute of Psychology Ruprecht‐Karls‐University of Heidelberg Heidelberg Germany; ^6^ Department of Social Work, Education, and Nursing University of Applied Sciences Esslingen Esslingen Germany

**Keywords:** individual therapy, mindfulness, relaxation, therapeutic alliance, youth

## Abstract

**Objectives:**

Mindfulness is increasingly being implemented in child and adolescent therapy. However, little is known about its effectiveness in individual settings within a clinical outpatient context. We investigated the effects of session‐introducing mindfulness interventions (IG‐M) compared to session‐introducing relaxation interventions (CG‐R) and no standardized session‐introduction (CG) on symptomatic outcome and therapeutic alliance in cognitive‐behavioral therapy using a randomized controlled design.

**Method:**

One thousand four hundred and seventy‐eight of 90 outpatient adolescents (11–19 years) diagnosed with depression, an anxiety disorder, or a hyperkinetic disorder were assessed. Symptomatic outcome was assessed by the German version of the Youth Self‐Report 11‐18R at five measurement time points (pre, 3rd, 10th, 17th therapy session, post), therapeutic alliance was assessed by the German version of the Therapeutic Alliance Scales for Children after every therapy session (1st–24th therapy session). Statistical analyses were conducted using multilevel modeling. The RCT was registered at ClinicalTrials.gov (NCT04034576).

**Results:**

Multilevel modeling revealed a significant decrease of symptomatic outcome in IG‐M compared to CG, but not compared to CG‐R, over time. A significant increase in therapeutic alliance over time was observed from the therapists' perspective with no group differences. Therapeutic alliance from patients' perspective significantly increased in IG‐M and significantly decreased in CG. There was no statistically meaningful change in therapeutic alliance from the patients' perspective in CG‐R.

**Conclusion:**

IG‐M had positive effects on symptomatic outcome and therapeutic alliance compared to CG, but did not outperform CG‐R. Future studies that control confounding variables are highly relevant to understand the role of mindfulness in individual youth psychotherapy.

## 1 Introduction

### 1.1 Definition of Mindfulness and Its Application in Psychotherapy

Mindfulness is defined as a specific form of attention that is non‐judgmental, purposeful, and focused on the present moment (Kabat‐Zinn 2013). Originally rooted in Buddhist traditions, mindfulness‐based interventions (MBIs) have increasingly been integrated into cognitive‐behavioral therapy (CBT) as part of the “third wave” (Hayes 2004; Perkins et al. 2023). The most well‐known psychotherapeutic applications are highly structured group programs (e.g., ‘Mindfulness‐Based Stress Reduction', MBSR; Kabat‐Zinn 2013, ‘Mindfulness‐Based Cognitive Therapy', MBCT; Segal et al. 2012, and its adaptations for children: MBCT‐C, Semple and Lee 2011; MBSR for school‐age children, Saltzman 2014). Other treatment packages that include MBIs are dialectical behavioral therapy (DBT; Linehan 1993) and acceptance commitment therapy (ACT; Hayes 2004). However, mindfulness interventions are often also used outside of treatment packages as single exercises in individual psychotherapy (Michalak et al. 2020).

### 1.2 Pre‐ and In‐Session Mindfulness Interventions

MBIs as a pre‐session intervention have mainly been implemented on therapists' side, with the aim of investigating the effects on therapeutic outcomes when therapists engage in a mindfulness exercise before the start of the therapy session with their patients (Dunn et al. 2013; Hunt et al. 2022; Mander et al. 2019). Results of these studies were inconclusive, ranging from no effects of pre‐session therapists' MBI on patients' outcome and on therapeutic alliance (Mander et al. 2019) to higher therapists' ratings of session effectiveness (Hunt et al. 2022) and higher therapist‐perceived therapeutic presence as well as higher patient‐perceived session effectiveness (Dunn et al. 2013).

To our knowledge, only one study investigated the effects of integrating mindfulness exercises in individual therapy in adult outpatients (Mander et al. 2019). More precisely, the study examined the effects of a 5‐min session‐introducing mindfulness intervention in contrast to a 5‐min session‐introducing relaxation intervention and no session‐introducing intervention within CBT therapy on symptomatic outcome and therapeutic alliance in 162 adult outpatients (Mander et al. 2019). No differences between the three conditions were found (Mander et al. 2019).

A recent meta‐analysis that explored the effects of both pre‐ and in‐session mindfulness exercises implemented in a psychotherapeutic context with adult patients detected, in addition to the already mentioned quantitative study by Mander et al. (2019), two further qualitative studies addressing in‐session mindfulness interventions (Goldberg et al. 2023). While dyads of trainee therapists (from a marriage and therapy training program) and patients qualitatively reported mainly positive effects of in‐session mindfulness exercises on therapeutic processing incorporated in individual psychotherapy (Horst et al. 2013), another study with adults in CBT‐informed treatment for managing seizures qualitatively reported no positive effects of in‐session mindfulness exercises on self‐efficacy and mastery (Michaelis et al. 2018).

In sum, the effects of single mindfulness exercises on patients' outcomes in individual psychotherapy remain largely unexplored, despite its frequent use by therapists (Michalak et al. 2020). With regard to younger age groups, to our knowledge, no study, neither qualitative nor quantitative, has investigated the effects of single in‐session mindfulness exercises yet.

### 1.3 Effects of Mindfulness on Symptomatology

Meta‐analyses indicate that MBIs are particularly effective for depression and anxiety in adults (Blanck et al. 2018; Goldberg et al. 2022). In children and adolescents, MBI effects are smaller but significant for mindfulness and anxiety/stress symptoms compared to active control conditions (Dunning et al. 2022). Studies also suggest positive effects in children with anxiety disorders (Borquist‐Conlon et al. 2019) and depression (Reangsing et al. 2021), while evidence for children with hyperkinetic disorders remains inconclusive (Evans et al. 2018; Lee et al. 2022).

A study on the effectiveness of the MBCT‐C program (Semple and Lee 2011) in juvenile patients with mental disorders proved that the MBCT‐C program was effective for adolescent patients diagnosed with an anxiety disorder and a comorbid bipolar disorder (Cotton et al. 2016; Cotton et al. 2020) and in youth with residual depressive symptoms after psychological treatment for a mood or an anxiety disorder (Ames et al. 2014). However, most studies investigating the effects of MBIs in children and adolescents, and, more specifically, the effectiveness of the MBCT‐C program, were conducted in school or non‐to‐subclinical settings rather than therapeutic settings (Carsley et al. 2018; McKeering and Hwang 2019).

### 1.4 Mindfulness and Therapeutic Alliance

MBIs may also enhance the therapeutic alliance, as mindfulness fosters self‐compassion and empathy which are both important factors for a sustainable patient‐therapist‐relationship (Kristeller and Johnson 2005). It can thus be assumed that the successful implementation of MBIs improves therapeutic alliance. Qualitative studies report positive effects of in‐session mindfulness practice on the patient‐therapist relationship in adults (Horst et al. 2013; Konichezky et al. 2022), whereas quantitative studies rather do not affirm this association (Garrote‐Caparrós et al. 2022; Mander et al. 2019). However, no study has investigated the effects of MBIs on therapeutic alliance yet.

### 1.5 Aims of the Present Study

At this point in time, it is unclear whether single in‐session mindfulness interventions yield positive effects on therapeutic outcomes in outpatient children and adolescents. Furthermore, very little is still known about the potential advantages of mindfulness interventions compared to other commonly used therapeutic interventions, for example, relaxation interventions (Mander et al. 2019).

For this reason, we aimed to close this research gap by designing an RCT that investigated the impact of single session‐introducing mindfulness exercises on the therapeutic process in individual child and adolescent CBT under routine care conditions in an outpatient training center. All mindfulness exercises were extracted from MBCT‐C program (Semple and Lee 2011), were adapted for individual therapy, and tested for feasibility in children and adolescents who attended outpatient psychotherapy (Kalmar et al. 2020). Single mindfulness exercises were integrated at the beginning of the routine therapy session so that patients did not receive extra treatment time when practicing mindfulness, in order to avoid confounding the potential effects of the mindfulness exercises with extra therapy time (Britton et al. 2018). As comparison conditions to the mindfulness condition (index group—mindfulness; IG‐M) served a relaxation condition as active control condition (active control group—relaxation; CG‐R), comprising traditional, session‐introducing relaxation interventions, and a standard treatment condition with no session‐introducing interventions (CG).

In line with the aforementioned findings, we hypothesized that IG‐M has positive effects on symptomatic outcome (Hypothesis 1) and therapeutic alliance (Hypothesis 2) over time in comparison to CG‐R and CG in outpatient children and adolescents.^1^

## 2 Method

### 2.1 Participants

The study was carried out between February 2019 and May 2024 at an outpatient training center specialized in child and adolescent CBT. The training fulfilled all standardized guidelines of CBT training in Germany. All participating trainee therapists held a Master's degree in either psychology or pedagogy, and completed an 18‐month internship in psychiatric and psychosomatic clinics before treating patients in the outpatient training center. Additionally, all participating therapists received at least 200 h of theory concerning the psychotherapeutic treatment of children and adolescents and a minimum of 60 h of self‐experience prior to entering the study.

Before treatment, juvenile patients were randomized to either IG‐M, CG‐R, or CG. The inclusion criteria for the patients were a primary diagnosis of a hyperkinetic disorder, a depressive disorder, or an anxiety disorder. Exclusion criteria referred to (1) an age below 11 and over 19 years, (2) deficient German language skills, (3) comorbid psychotic disorder, and (4) acute suicidality. Other comorbidities of the patients were not necessarily categorized as long as a hyperkinetic disorder, a depressive disorder or an anxiety disorder was the main diagnosis. Drop‐out criteria were fulfilled if a patient (a) terminated individual psychotherapy before the 24th therapy session against therapeutic advice, (b) refused further study participation, (c) was not able in taking part in the scheduled study interventions. In total, 90 patients with 38 therapists were included in the study. Twenty‐nine patients were allocated to the IG‐M, 30 patients were allocated to the CG‐R, and 31 patients were allocated to the CG. In IG‐M, seven patients dropped‐out prior to post‐assessment due to insufficient treatment motivation or therapist change. In CG‐R, nine patients dropped out due to lack of motivation to engage in relaxation exercises, relocation to another city, severe symptomatology, and an extended stay abroad. In CG, in total of seven patients dropped out due inpatient hospitalization, burden caused by the assessments after every therapy session, and greater motivation to engage in hobbies than in therapy. In total, 86, patients provided at least one assessment at one of the measurement time points and were therefore included in the statistical analyses (see Figure 1, CONSORT flow diagram).

**Figure 1:**
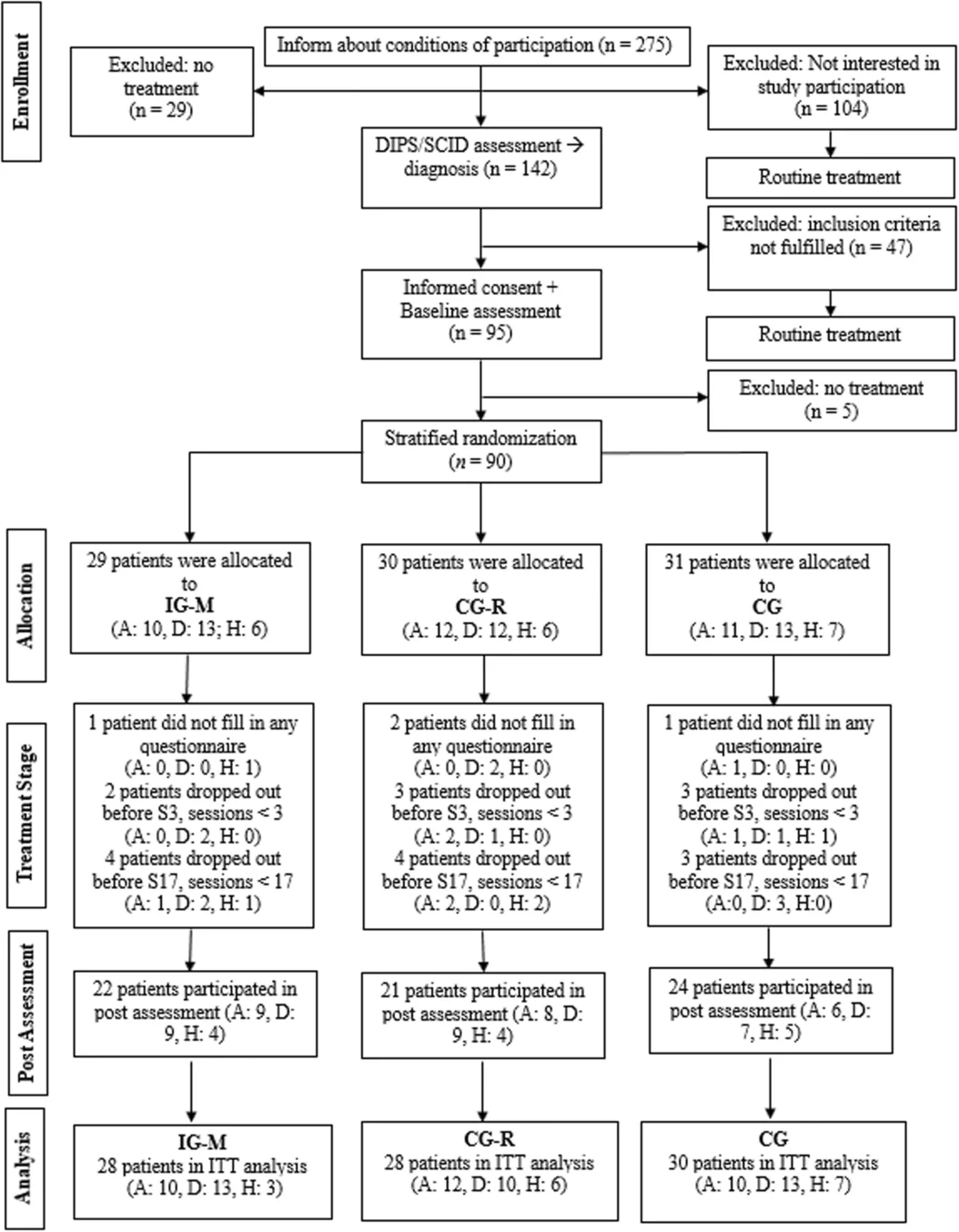
CONSORT Flow Diagram. A, anxiety; CG, passive control group; CG‐R = control group ‐ relaxation; CG‐R, control group ‐ relaxation; D, depression; H, hyperkinetic disorder; ITT, intention‐to‐treat; IG‐M, intervention group ‐ mindfulness; S17, 17th session; S3, third session.

Thirty‐eight patients were diagnosed with a depression, 33 patients were diagnosed with an anxiety disorder, and 19 patients were diagnosed with a hyperkinetic disorder. Among the depressive disorders, moderate depressive disorder was the most common diagnosis, accounting for over 50% of the depressive disorders. Other diagnoses in the depressive group were mild depressive disorder, recurrent depressive episode, dysthymia, adjustment disorder with mainly depressive symptoms, acute stress reaction with additional depressive symptoms, mixed anxiety and depressive disorder with mainly depressive symptoms, and other childhood emotional disorders with mainly depressive symptoms. Concerning the hyperkinetic disorders, the combined type of attention‐deficit/hyperactivity disorder (ADHD) was the most common diagnosis with over 60%. Otherwise, the diagnoses in the hyperkinetic group consisted of the predominantly inattentive type of ADHD, oppositional defiant disorder with additional hyperactive/impulsive symptoms, and other mixed diagnoses of conduct and emotions with additional hyperactive/impulsive symptoms. With regard to the anxiety disorders, the most common diagnosis was social phobia, accounting for over 40% of the anxiety disorders. Other diagnoses in the anxiety group were agoraphobia, specific phobia, generalized anxiety disorder, posttraumatic stress disorder, separation anxiety disorder, mixed anxiety and depressive disorder with mainly anxious symptoms, adjustment disorder with mainly anxious symptoms, and other childhood emotional disorders with mainly anxious symptoms. For more sample characteristics, see Table 1.

**Table 1:** Baseline^a^ characteristics^b^ of the participating^c^ patients and therapists.

Characteristic	Full sample (*n* = 90)	IG + M (*n* = 29)	CG + R (*n* = 30)	CG (*n* = 31)	*p*
Age, *M (SD)*	15.11 (2.50)	15.90 (2.50)	14.77 (2.37)	14.71 (2.53)	0.12^a^
Sex					0.93^b^
Female, *n* (%)	67 (74.44)	21 (72.41)	23 (76.67)	23 (74.19)	
Male, *n* (%)	23 (25.56)	8 (25.56)	7 (23.33)	8 (25.81)	
Educational level					0.76^c^
Comprehensive school, *n* (%)	6 (6.67)	1 (3.45)	2 (6.67)	3 (9.68)	
Low to middle educational level, *n* (%)	21 (23.33)	8 (27.59)	6 (20)	7 (22.58)	
High educational level, *n* (%)	48 (53.33)	13 (44.83)	18 (60)	17 (54.84)	
University/Vocational education, *n* (%)	8 (8.89)	4 (13.79)	1 (3.34)	3 (9.68)	
Others, *n* (%)	7 (7.78)	3 (10.34)	3 (10)	1 (3.22)	
Main diagnosis					0.99^b^
Depression, *n* (%)	38 (42.22)	13 (44.83)	12 (40)	13 (41.94)	
Anxiety, *n* (%)	33 (35.56)	10 (34.48)	12 (40)	11 (35.48)	
Hyperkinetic disorder, *n* (%)	19 (22.22)	6 (20.69)	6 (20)	7 (22.58)	
Additional comorbid diagnosis (One or More), *n* (%)	36 (40.00)	11 (37.93)	13 (43.33)	13 (41.94)	0.95^b^
Receiving psychopharmaceuticals, *n* (%)	24 (26.67)	9 (31.03)	10 (33.33)	5 (16.13)	0.26^b^
Therapists (*n* = 38)					
Female sex, *n* (%)		38 (100)			
Age, *M (SD)*		28.58 (3.24)			
Treated Outpatients before Study Entry, *M (SD)*		3.61 (5.77)			
Conducted Therapy Sessions before Study Entry, *M (SD)*		52.08 (112.99)			

*Note:* School type: comprehensive school, 9–12/13 years of education, based on pupils' individual capabilities; low to middle educational level, 9–10 years of education, terminating with a lower or intermediate secondary school leaving certificate; high educational level, 12–13 years of education, qualifying for university entrance; others: Waldorf education/Montessori educational theory, elementary school, special needs school, not further specified.

Abbreviations: CG, passive control group; CG‐R, active control group – relaxation; IG‐M, intervention group ‐ mindfulness; M, Mean; SD, Standard Deviation.

^a^ Analysis of variance.

^b^ *χ*2 test.

^c^ Fisher's exact test (conducted if at least one cell of the analysis had an expected frequency lower than 5).

### 2.2 Measures

#### 2.2.1 Diagnoses

Diagnoses of mental disorders for patients under 18 years were assessed by means of the child and adolescent version of the Diagnostic Interview for Mental Disorders—Open Access (Kinder‐DIPS; Margraf et al. 2017; Schneider et al. 2017) according to the German Version of the ICD‐10 (World Health Organzation 2019) and DSM‐5 (American Psychiatric Association 2013) criteria. Diagnoses for patients aged 18 years or older were made according to the Structured Clinical Interview (SCID; First et al. 2015). Video recording of the diagnostic interviews of the patients took place in 35 cases. A second rating of 65.71% of the diagnostic videos (23 videos) was randomly selected. In 47.83% of cases, the second rating confirmed the given primary diagnosis, but differed regarding the number of reported symptoms. However, the threshold for the corresponding primary diagnosis has been reached in all cases. In the remaining 52.17% of cases, the ratings were in complete agreement with the primary diagnosis. No other diagnostic information (e.g., specific symptom profiles taking comorbid diagnoses into account) were checked by the second rating. This verification procedure assessed the agreement regarding the primary diagnostic classification associated symptom counts only.

#### 2.2.2 Primary Outcome Measures

The German version (Döpfner et al. 2014) of the Youth Self‐Report 11‐18 R (Achenbach and Rescorla 2001) is a frequently used measure to assess general psychopathological symptomatology in juvenile patients. It consists of 119 items that form eight syndrome scales, three broad band scales, and a subscale measuring social desirability. The eight syndrome scales comprise “anxious/depressed,” “withdrawn/depressed,” “somatic complaints,” “social problems,” “thought problems,” “attention problems,” “rule breaking behavior,” and “aggressive behavior,” and the three broad‐band scales include “internalizing problems,” “externalizing problems,” and the “total score.” All items were rated on a three‐stepped scale from 0 (*not true*) to 2 (*very true or often true*). Higher scores point towards more psychopathological symptomatology. The YSR 11‐18R total score represents a measure of a common factor of psychopathology in youth (‘*p*’*‐*factor; Achenbach et al. 2025). This *p*‐factor has recently gained increasing attention in research as it is claimed to be a better predictor for maladaptation than disorder‐specific assessments (Caspi and Moffitt 2018).

We used multilevel McDonald's *ω* (Gomez et al. 2014) to estimate the reliability of within‐person variation in the primary outcome measures in our sample. Internal consistency ranged between 0.68 (thought problems) and 0.89 (aggressive behavior) on the within‐person and 0.24 (aggressive behavior) and 0.89 (anxious/depressed) on the between‐person level for the eight syndrome scales of the YSR 11‐18R.

#### 2.2.3 Secondary Outcome Measures

Therapeutic alliance was assessed with the German version of the Therapeutic Alliance Scales for Children (FTB‐KJ; Kronmüller et al. 2003; Shirk and Saiz 1992). The questionnaire can be administered to both patients and therapists. Both FTB‐KJ versions (patient version, therapist version) consist of 12 items and are rated on a four‐step scale from 1 (*not true at all*) to 4 (*exactly true*). It comprises the three subscales “bond,” “negativity,” and “verbalization,” that can be integrated in an alliance total score (Kronmüller et al. 2003). Internal consistencies in the present sample were *ω*_within_ = 0.82 and *ω*_between_ = 0.91 for the patient perspective, and *ω*_within_ = 0.88 and ω_between_ = 0.88 for the therapist perspective. Correlations between the two FTB‐KJ versions are moderate. The FTB‐KJ correlates positively with other instruments that assessed therapeutic alliance (Kronmüller et al. 2003). With regard to symptomatology, the FTB‐KJ patients' version correlates negatively with symptom severity, most notably in boys (Kronmüller et al. 2003).

#### 2.2.4 Demographic Data, Adherence, In‐Session Therapeutic Presence, Therapeutic Techniques, Allegiance

Standard demographic data of the juvenile patients were assessed (e.g., age, sex, school). Additionally, information about former experiences with mindfulness and relaxation of the children and adolescents was collected. Parents of the participating patients gave information about medication intake.

Additionally, adherence, in‐session therapeutic presence, therapeutic techniques, and allegiance were assessed. Data were collected through questionnaires and observer‐based ratings. In case of observer ratings, ratings were conducted by psychologists holding a Master's level degree and were trained in the particular assessment by the principal investigators of the study. For a detailed overview of the measures used, see Appendix B.

### 2.3 Procedures

The present study was a three‐arm randomized controlled trial, conducted at a single center. It was registered at ClinicalTrials. gov (NCT04034576). Detailed information about the study protocol was published elsewhere (Kalmar et al. 2022). The study design comprised 24 therapy sessions of child and adolescent psychotherapy, with a length of typically 50 min each session. Diagnoses and diagnosis severity were assessed before (pre) and after the study (post). Self‐ratings of the patients concerning their clinical symptoms, mindfulness, and self‐compassion were assessed at pre, after the 3rd therapy session (S3), after the 10th therapy session (S10), after the 17th therapy session (S17), and after the 24th therapy session (S24). Additionally, patients' adherence, perceived therapeutic presence, perceived quality of the session‐introducing interventions, and allegiance effects were assessed at S3, S10, S17, and S24. Parallel to the patients' assessment, data of the therapists regarding therapeutic presence, therapeutic techniques, adherence, and allegiance was assessed at pre, S3, S10, S17, and S24. Therapeutic alliance of both patients and therapists was assessed after each therapy session (S1 to S24). All therapy sessions were recorded and trainee therapists had regular meetings (on average after every fourth therapy session) with experienced therapists (supervisors) to review the therapy course. Supervisors were licensed child and adolescent psychotherapists who had at least 5 years of work experience in the treatment of children and adolescents, as well as 3 years of work experience in the teaching of trainee psychotherapists.

Participant recruitment began with a telephone screening for symptomatology. Eligible patients and their caregivers were then invited for an in‐person meeting with a study trainee therapist, who explained the study and conducted a semi‐structured diagnostic interview. Participation was voluntary, requiring written informed consent from both patients and caregivers. The allocation of the patients to one of the three treatment arms was conducted by a blocked and stratified randomization process with a computerized random number generator. Patients were stratified according to their main diagnosis (anxiety disorder, depressive disorder, hyperkinetic disorder). Equal randomization of the patients to one of the three treatment arms was accomplished by a balanced blocking procedure. An independent research assistant that was not part of the study team was in charge of the randomization process, as postulated in the CONSORT statement (Altman et al. 2001). No other person had access to the randomization process. The aim of the present study was to generate results that generalize to routine clinical practice; hence the study focus was on external validity. For this reason, in line with the extension of the CONSORT statement regarding the criteria for a pragmatic RCT (Zwarenstein et al. 2008), neither the patients nor the trainee therapists were blinded to the treatment conditions. However, although aims and methodology of the study was known to the study participants, they had no knowledge about the specific study hypotheses. We applied a crossed therapist design in which a given therapist delivered the experimental as well as the control conditions. This crossed therapist design allows addressing the issue of potential therapist effects. More specifically, as efficacy varies across therapists, a crossed therapist design allows controlling for these effects as the same therapists perform all different treatment conditions. Both the mindfulness and the relaxation interventions were introduced in a neutral way and preparation in relaxation techniques was as intensive as mindfulness preparation. The study protocol was approved by the university's internal review board in accordance with the Helsinki Declaration. For more information, see the CONSORT flow diagram in Figure 1.

### 2.4 Interventions

#### 2.4.1 Workshop Training

Trainee therapists participating in the study were obliged to take part in two mindfulness workshops including a 6‐week self‐experience interval. The structure of the mindfulness workshops and of the 6‐week self‐experience interval is based on a mindfulness training that was successfully implemented in CBT training by Mander et al. (2017). The workshops were held by a mindfulness expert with over 20 years of experience in the mindfulness area. During the first workshop, trainee therapists learned about the theoretical background of mindfulness, its integration into third‐wave approaches of CBT, and its adaptation to child and adolescent psychotherapy (with special focus on the MBCT‐C program; Semple and Lee 2011). Additionally, trainee therapists experienced by themselves formal and informal mindfulness practices, instructed by the mindfulness expert. During the 6‐week self‐experience interval, trainee therapists had the task to carry out mindfulness practices by themselves (both formal and informal) at home. During the second workshop, experiences of the trainee therapists with regard to the self‐experience interval were discussed and the mindfulness practices for children and adolescents of the present study were introduced. Both workshops including the self‐experience interval mainly received positive feedback (for more information see Kalmar et al. 2024).

Besides the mindfulness training, all participating trainee therapists received two workshops focusing on relaxation techniques such as imaginative techniques, progressive muscle relaxation (PMR), and autogenic training. The workshops were part of the regular trainee therapist program and took place before the start of the study. As in the mindfulness workshops, therapists were asked to practice relaxation at home between the workshops.

#### 2.4.2 Routine Therapy

All patients received routine CBT at a university outpatient clinic, including psychoeducation, cognitive restructuring, exposure, and creative child psychotherapy techniques (Stallard et al. 2014). Patients of all groups received routine CBT, differing only in the first 5–10 min for the intervention. However, CBT treatment was individualized according to patients' primary diagnoses (depressive disorder, anxiety disorder, or hyperkinetic disorder) and needs. Disorder‐specific treatment followed the current German clinical practice guidelines for mental disorders (Deutsche Gesellschaft für Kinder‐ und Jugendpsychiatrie, Psychosomatik und Psychotherapie DGKJP 2013; Deutsche Gesellschaft für Kinder‐ und Jugendpsychiatrie, Psychosomatik und Psychotherapie (DGKJP) et al. 2018; Deutsche Gesellschaft für Psychosomatische Medizin und Ärztliche Psychotherapie DGPM 2021; Schulte‐Körne et al. 2023). For example, the main focus in the treatment of juvenile depression is on identifying typical depressive thought patterns (Beck 1967) whereas the main focus in treating juveniles with hyperkinetic disorders is on time management, task planning, and emotion regulation (Caye et al. 2019). In the treatment of juvenile anxiety, exposure‐based interventions, alongside with cognitive restructuring, occupy a central role (Wehry et al. 2015).

#### 2.4.3 Intervention Implementation

Session‐introducing interventions (IG‐M, CG‐R) were implemented by the therapists at the beginning of the first 24 therapy sessions. The interventions were delivered by a block‐wise algorithm: four times intervention A (IG‐M: ‘mindful walking', CG‐R: ‘walking relaxation'), four times intervention B (IG‐M: ‘body scan', CG‐R: ‘PMR'), four times intervention C (IG‐M: ‘breathing space', CG‐R: ‘imagery journey'). Then the procedure was repeated for the next 12 therapy sessions. For an overview of the intervention implementation, see Figure 2.

**Figure 2:**
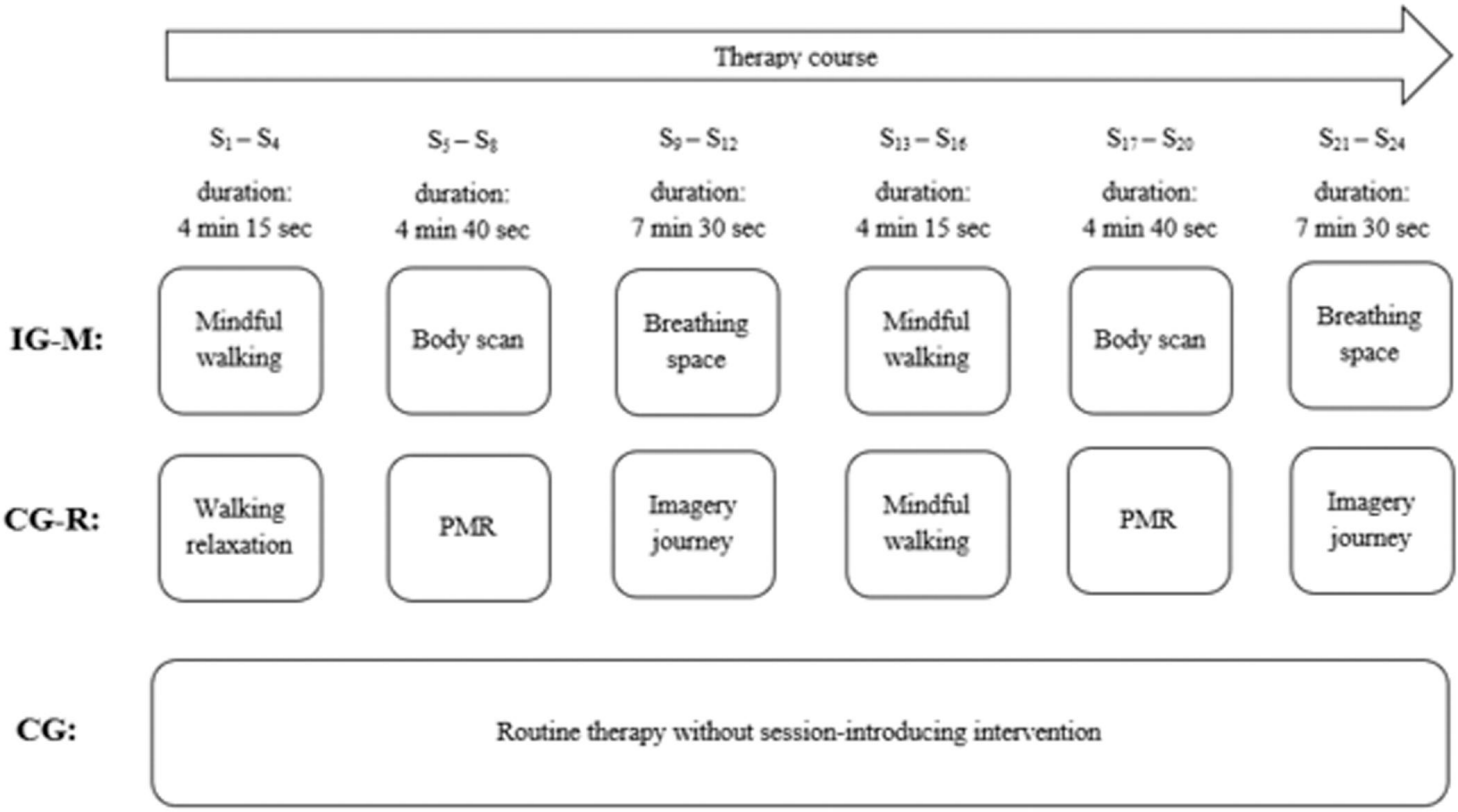
Intervention implementation. CG, control group; CG‐R, control group‐relaxation; IG‐M, intervention group‐mindfulness; PMR, progressive muscle relaxation; S, therapy session.

Before the first intervention, patients were instructed by their therapists about the clinical relevance of their study condition with its interventions in a standardized way (pre‐formulated wording). After each intervention, the therapists conducted an inquiry and reflected together with their patients on the experiences during the interventions.

#### 2.4.4 Intervention Group—Mindfulness (IG‐M)

In IG‐M, each session began with a 5–10 min mindfulness intervention adapted from the MBCT‐C program (Semple and Lee 2011). Three interventions were used: “mindful walking” (4 min 15 s), “body scan” (4 min 40 s), and “breathing space” (7 min 30 s). All interventions were adapted by JK and JM and assessed for suitability by 10–12 juvenile outpatients (Kalmar et al. 2020). See Appendix C for the English translation of the three mindfulness interventions.

#### 2.4.5 Active Control Group—Relaxation (CG‐R)

In CG‐R, each session began with a 5–10 min relaxation intervention, designed to parallel the mindfulness interventions in duration and theme. In collaboration with an experienced child and adolescent psychotherapist, the authors developed a “walking relaxation” exercise (4 min 15 s) as a counterpart to “mindful walking.” The counterpart to the “body scan” was a “PMR” exercise (4 min 40 s; Speck, 2013). The final intervention, parallel to “breathing space,” was an adapted imagery journey called “dolphin riding” (7 min 30 s; Petermann et al. 2018). All interventions were adapted by JK and JM and assessed for suitability by 10–12 juvenile outpatients (Kalmar et al. 2020). See Appendix C for the English translation of the three relaxation interventions.

#### 2.4.6 Passive Control Group—Treatment‐as‐Usual (CG)

No session‐introducing intervention was conducted in CG. Therapists had no restrictions how to start the therapy session with their patients.

### 2.5 Statistical Analyses

Power analyses with G*Power (Faul et al. 2007) for the detection of an effect of Cohen's *f* = 0.12 for the interaction between time (pre, S3, S10, S17, S24) and treatment condition (IG‐M vs. CG‐R vs. CG; analysis of variance (ANOVA) repeated measures, within‐between‐interaction, *α* = 0.05, power (1 − β) = 0.80, number of groups = 3, number of measurements = 5, pre–post correlation *r* = 0.5, nonsphericity correction = 1) for the primary outcome (total score of the YSR 11‐18R) resulted in a sample size of 108 patients. While this serves only as an approximation of the power of the reported multilevel models (MLMs), previous research has demonstrated that the power of the MLMs is often similar to, or even greater than, that of an ANOVA design (Baldwin et al. 2014). Consequently, the planned sample size should be viewed as a conservative estimate necessary to detect a condition x time interaction.

We investigated changes in general psychopathological symptomatology and therapeutic alliance using MLMs. Specifically, we used three‐level models, in which repeated assessments (Level 1) were nested within patients (Level 2), which were nested in therapists (Level 3). Level 1 pseudo R^2^ according to Xu (2003) which represents the amount of Level 1 variance accounted for by the model (i.e., the amount of within‐patient fluctuations in psychopathological symptomatology/therapeutic alliance i.e. explained by the predictors included in the model), was used as a measure of effect size. To test our hypothesis whether patients of IG‐M will improve more in psychopathological symptomatology compared to patients in CG‐R and in CG, we predicted psychopathological symptomatology obtained at pre, S3, S10, S17, and S24 from treatment condition, and time (primary analysis). The condition variable was added by two dummy variables that compared (1) IG‐M to CG‐R and (2) IG‐M to CG. In line with prior empirical work (Mander et al. 2019), time was coded as 1, 2, 3, 4, and 5, and logarithmized before being added to the model. We controlled for patients' age, which was centered on the sample mean, gender, comorbidity, and disorder group. While gender and comorbidity were entered as dichotomous variables, we added disorder group by dummy variables that compared anxiety disorder with depressive disorder, and anxiety disorder with hyperkinetic disorder. To test our prediction that patients and therapists of IG‐M will experience greater increases of therapeutic alliance over the course of therapy in comparison to CG‐R and CG (secondary analysis), we used a multivariate MLM (Baldwin et al. 2014; Mander et al. 2019) allowing us to estimate trajectories of patients' ratings and therapists' ratings simultaneously. To this end, we created a dichotomous variable (perspective) which indicates whether the rating was based on the therapists' perspective (*perspective* = 0) or on the patients' perspective (*perspective* = 1). Additionally, the continuous session number that ranged from S1 to S24 was log transformed before the analyses and then added as the predictor time. Perspective, condition (coded as in the model described above), and time were added as predictors. We controlled for the same covariates as in the model described above. In the second model, we added the two‐way interactions among the predictors perspective, condition, and time. In the last model, we additionally added a three‐way interaction perspective x condition x time. In case of a significant interaction, this model was followed up by two models that separate the changes into patients' and therapists' perspective (post‐hoc tests). In the presence of an additional interaction effect in one or both of these two models, we conducted simple slope analyses to disentangle the interaction effect across IG‐M, CG‐R, and CG conditions (post‐hoc tests). These follow‐up analyses were carried out to facilitate the interpretation of significant interactions. No correction for multiple testing was applied to these simple slope analyses.

Finally, according to the guidelines of Jacobson and Truax (1991), we calculated response and remission rates for patients and compared them across IG‐M, CG‐R, and CG (secondary analysis). Patients were considered responders if they achieved a reliable change index (RCI) of ≥1.96. Those who also met the YSR 11‐18R cut‐off for non‐pathological symptomatology were categorized as remitters.

Statistical test families consist of symptomatology‐related models (Hypothesis 1), therapeutic alliance‐related models (Hypothesis 2), and information about response and remitter rates. Statistical analyses were performed using the statistical program R (version 4.3.1; R Core Team 2023) running RStudio (version 2024.12.0; Posit team 2024). MLMs were conducted using the R package nlme (version 3.1.−167; Pinheiro et al. 2025).

### 2.6 Deviations from the Study Protocol

Power analyses with G*Power (Faul et al. 2007) resulted in a sample size of 108 patients. To account for 25% dropout, we had planned with a sample size of 135 patients (Kalmar et al. 2022). However, due to recruitment challenges (SARS‐CoV‐2 pandemic and parents expressing concerns about their children participating in scientific studies), only 90 patients were assigned to the three treatment arms. Additionally, we stipulated a third hypothesis in our study protocol that IG‐M would moderate the association between therapeutic alliance and symptomatology. Due to the smaller than intended sample size, analyses regarding this hypothesis are most likely underpowered. We therefore decided to not include the results of the third hypothesis in the main manuscript. In the interest of full transparency, results are reported in Appendix A. No other deviations from the study protocol occurred.

## 3 Results

### 3.1 Descriptive Analyses

A total of 1478 therapy sessions were assessed. Baseline characteristics were similar across IG‐M, CG‐R, and CG (see Table 1). Of the 90 patients, 69 provided prior mindfulness or relaxation experience details: 78.3% had mindfulness experience, and 79.7% had relaxation experience. No significant differences were found between groups for prior experience (Fisher's exact test *p* = 0.48 for mindfulness, *p* = 0.23 for relaxation). Main comorbidities included anxiety in depressive patients (21.05%), and depression in anxious (30.30%) and hyperkinetic patients (15.79%). No race/ethnicity data were collected due to the sensitivity of these data in German history (Boytchev 2023). Therapies were conducted by 38 trainee therapists, with 82% having mindfulness experience and 58% having relaxation experience prior to the workshops. For means, standard deviations and effect sizes of symptomatology and therapeutic alliance, see Table 2. To contextualize the mean level changes over time, pre‐post effect sizes for symptomatic outcome and therapeutic alliance were calculated using Cohen's *d*_*z*_ for dependent samples, based on the standard deviation of the difference scores within the three groups (see Table 2; see following sections for model‐based effect sizes for group differences in change over time).

**Table 2:** Sum score/means and standard deviations (in parentheses) of YSR 11‐18R, FTB‐KJ (patient), and FTB‐KJ (therapist).

	Entire Sample	IG ‐ M	CG‐R	CG
*YSR 11‐18 R (Sum Score)*				
Pre	56.00 (25.22)	59.04 (25.12)	58.28 (24.36)	51.67 (26.23)
Session 3	60.22 (25.91)	58.45 (22.58)	64.59 (27.41)	57.21 (28.36)
Session 10	54.93 (28.69)	53.53 (23.55)	54.53 (31.41)	56.94 (32.19)
Session 17	52.51 (29.50)	43.55 (22.33)	64.63 (31.18)	51.53 (33.25)
Session 24	50.00 (31.80)	42.94 (21.51)	56.15 (33.10)	53.08 (41.30)
Effect size [95% CI]	0.41 [0.09, 0.74]	0.73 [0.14, 1.29]	0.63 [‐0.03; 1.27]	0.05 [‐0.50; 0.59]
*FTB‐KJ, Patient (Mean)*				
Session 1‐3	3.52 (0.34)	3.52 (0.30)	3.47 (0.37)	3.58 (0.34)
Session 4‐10	3.54 (0.37)	3.61 (0.29)	3.45 (0.42)	3.54 (0.38)
Session 11‐17	3.48 (0.50)	3.70 (0.23)	3.43 (0.44)	3.33 (0.67)
Session 18‐24	3.56 (0.42)	3.75 (0.22)	3.35 (0.49)	3.56 (0.43)
ES [95% CI]	−0.12 [−0.39, 0.15]	−0.81 [−1.31, −0.29]	0.18 [−0.28; 0.63]	0.35 [−0.18; 0.86]
*FTB‐KJ, Therapist (Mean)*				
Session 1–3	3.04 (0.41)	3.26 (0.30)	2.90 (0.39)	2.97 (0.44)
Session 4–10	3.14 (0.38)	3.28 (0.30)	3.08 (0.40)	3.06 (0.41)
Session 11–17	3.15 (0.50)	3.40 (0.34)	2.95 (0.47)	3.09 (0.56)
Session 18–24	3.25 (0.44)	3.44 (0.37)	3.00 (0.47)	3.31 (0.35)
ES [95% CI]	−0.42 [−0.70, −0.14]	−0.43 [−0.89, 0.05]	−0.33 [−0.79; 0.14]	−0.57 [−1.09; −0.03]

*Note:* CG‐R sample, 13 ≤ *n* ≤ 25; CG sample, 13 ≤ *n* ≤ 30. Entire sample, 43 ≤ *N* ≤ 79; ES, Effect Size for the Pre‐Post‐Differences (Cohen's *d*_*z*_, for dependent samples, based on the standard deviation of the difference scores within the three groups); FTB‐KJ, Therapeutic Alliance Scales for Children, mean score for the four therapy segments (early therapy phase, mid‐therapy phase I, mid‐therapy phase II, late therapy phase; IG‐M, intervention group – mindfulness; IG‐M sample, 17 ≤ *n* ≤ 24; YSR 11 ‐18R, Youth Self‐Report 11‐18Revised.

Abbreviations: CG, passive control group; CG‐R, control group – relaxation; CI, Confidence Interval.

### 3.2 Treatment Integrity

Treatment integrity was assessed through adherence, allegiance, competence, and the use of CBT techniques. Treatment integrity was given, as indicated by various measurements (for more information, see Appendix B). However, group differences in two subscales (‘Exercising and Suggestive Interventions', ‘Interventions Based on Operant Conditioning and Transfer') of the Heidelberg Inventory of Cognitive‐Behavioral Interventions (HICBI; D'Errico et al. 2018) were detected. With regard to “Exercising and Suggestive Interventions,” therapists indicated that they applied more of these interventions in the IG‐M and CG‐R than in the CG. Concerning the subscale “Interventions Based on Operant Conditioning and Transfer,” higher values in the IG‐M in contrast to the CG‐R and CG were detected. For more information, see Appendix B.

### 3.3 Are Symptoms Reduced More Strongly in IG‐M Compared to CG‐R and CG?

We set up models of increasing complexity to test whether patients of IG‐M, CG‐R, and CG differed in their general psychopathological symptomatology across therapy. In the first empty model (see Model 0 in Table 3), we included random intercepts on the person (Level 2) and on the therapist (Level 3) level. The results showed that less than 1% of the variation in symptom severity was attributed to differences between therapists, and 77.6% to differences between patients. Next, we added the main effects of condition and (logarithmized) time, as well as the main effects of age, gender, comorbidity, and disorder group (see Model 1 in Table 3). Results of this model showed that psychopathological symptomatology decreased over time across all groups, *b* = −5.074, *p* = 0.004. The model explained 34.2% of the within‐patient variance in symptom severity. Last, we added the condition x time interaction to investigate whether the change in symptom severity differed between IG‐M, CG‐R, and CG (Model 2 in Table 3). This interaction explained 18.94% of the random slope variance of the time (i.e., group accounted for 18.94% of the between‐participant differences in change of symptomatology over time). Estimated marginal means of the model are reported in Table D1 (Appendix D), demonstrating a noticable decrease in IG‐M over time from 57.6 at pre to 39.2 at post, a smaller decrease in CG‐R from 59.9 at pre to 52.6 at post, and relatively stable values in CG with 51.0 at pre and 52.0 at post. Results showed a significant interaction for IG‐MvsCG x time, *b* = 12.035, *p* = 0.003. This means that psychopathological symptomatology in IG‐M decreased more strongly over time than in CG. However, changes in psychopathological symptomatology over time did not significantly differ between IG‐M and CG‐R, *b* = 6.898, *p* = 0.095.^2^ These results are therefore partially consistent with H1. The statistical test family of symptomatology‐related models consisted of a series of three, progressively more complex models due to the hierarchical structure of the data (main confirmatory tests). Model 0 and Model 1 were preceding, model‐building steps, and Model 2 was the final model to answer H1 (principal test of interest; interpretation of the two‐way interaction condition x time). No correction for multiple testing was applied.

**Table 3:** Results of MLM growth curve analyses: Fixed and random effect predictors of symptomatic outcome (Youth Self‐Report 11‐18 R, total score).

	Model 0		Model 1		Model 2	
Fixed effects						
Intercept	54.168***	(2.755)	31.466**	(11.906)	34.944**	(12.009)
Age	—		0.644	(1.297)	0.826	(1.302)
Gender^a^	—		7.608	(6.102)	7.607	(6.126)
Comorbidity^b^	—		12.178*	(5.283)	12.242*	(5.307)
ANXvsDEP	—		‐1.229	(7.071)	‐0.490	(7.101)
ANXvsHYPER	—		15.868*	(6.097)	15.469*	(6.123)
IG‐MvsCG‐R	—		6.057	(6.096)	2.315	(6.495)
IG‐MvsCG	—		‐0.659	(6.077)	‐6.605	(6.416)
Time	—		‐5.074**	(1.758)	‐11.408***	(2.850)
Time x IG‐MvsCG‐R	—		—		6.898	(4.110)
Time x IG‐MvsCG	—		—		12.035**	(4.031)
Random effects (variances)						
Intercept (Level 3)	0.000		0.000		0.000	
Time (Level 3)	—		0.000		0.000	
Intercept (Level 2)	583.959		431.639		426.162	
Time (Level 2)	—		108.169		87.677	
Residual Variance	168.290		110.812		109.623	
*R^2^* (Level 1)	—		34.15%		34.86%	
AIC	2534.283		2497.208		2492.384	

*Note:* Number of observations (Level 1) = 291, number of patients (Level 2) = 86, number of therapists (Level 3) = 37. Age was centered on the sample mean, time was coded 1–5, and log transformed before the analyses. The table depicts point estimates and standard errors in parentheses. Significant values are marked in bold. ^a^0 = female, 1 = male, ^b^0 = no comorbidity, 1 = comorbid disorder, *p < 0.05, **p < 0.01, ***p < 0.001.

Abbreviations: AIC, Akaike information criterion; ANX, anxiety disorder; ANXvsDEP, anxiety versus depressive disorder; ANXvsHYPER, anxiety versus hyperkinetic disorder; CG, passive control group; CG‐R, control group ‐ relaxation; DEP, depressive disorder; DEPvsHYPER, depressive versus hyperkinetic disorder; HYP, hyperkinetic disorder; IG‐M, mindfulness intervention group; IG‐MvsCG, mindfulness intervention versus passive control group; IG‐MvsCG‐R, mindfulness intervention versus control group ‐ relaxation.

### 3.4 Is There a Greater Increase in Therapeutic Alliance in IG‐M Compared to CG‐R and CG?

In the first model (see Model 0 in Table 4), we included the dichotomous predictor variable perspective as both a fixed and a random effect. Results showed that across all sessions and all groups, therapeutic alliance was rated higher by the patients than by the therapists, *b* = 0.395, *p* < 0.001. In the next model, we added the main effects for condition, (logarithmized) time, and the covariates age, gender, comorbidity, and disorder group (see Model 1 in Table 4). Results showed that therapeutic alliance significantly increased over time, *b* = 0.029, *p* = 0.031. Additionally, therapeutic alliance was rated significantly lower in CG‐R than in IG‐M condition, *b* = −0.195, *p* = 0.026.

**Table 4:** Results of MLM growth curve analyses: Fixed and random effect predictors of therapeutic alliance.

	Model 0	Model 1	Model 2	Model 3
Fixed effects				
Intercept	3.138*** (0.045)	3.032*** (0.337)	2.944*** (0.336)	2.997*** (0.336)
Age	—	‐0.002 (0.018)	0.000 (0.018)	0.000 (0.018)
Gender^a^	—	0.098 (0.086)	0.099 (0.085)	0.099 (0.085)
Comorbidity^b^	—	−0.055 (0.073)	−0.054 (0.072)	−0.055 (0.072)
ANXvsDEP	—	0.023 (0.097)	0.027 (0.096)	0.029 (0.096)
ANXvsHYPER	—	0.097 (0.087)	0.081 (0.086)	0.081 (0.086)
IG‐MvsCG‐R	—	−0.195* (0.084)	−0.187 (0.103)	−0.237* (0.109)
IG‐MvsCG	—	−0.145 (0.086)	−0.160 (0.105)	−0.289* (0.111)
Time	—	0.029* (0.013)	0.100*** (0.026)	0.060* (0.029)
Perspective^c^	0.395*** (0.045)	0.394*** (0.046)	0.359*** (0.075)	0.273*** (0.080)
Time x IG‐MvsCG‐R	—	—	−0.072* (0.029)	−0.039 (0.038)
Time x IG‐MvsCG	—	—	−0.067* (0.029)	0.018 (0.038)
Time x Perspective	—	—	−0.049* (0.020)	0.016 (0.029)
Perspective x IG‐MvsCG‐R	—	—	0.164 (0.085)	0.233* (0.098)
Perspective x IG‐MvsCG	—	—	0.225** (0.086)	0.408*** (0.100)
Time x Perspective x IG‐MvsCG‐R	—	—	—	−0.054 (0.038)
Time x Perspective x IG‐MvsCG	—	—	—	−0.140*** (0.039)
Random effects (variances)				
Intercept (Level 3)	0.003	0.005	0.018	0.019
Perspective (Level 3)	0.022	0.024	0.056	0.059
Time (Level 3)	—	0.001	0.005	0.005
Time x Perspective (Level 3)	—	—	0.004	0.004
Intercept (Level 2)	0.144	0.115	0.110	0.107
Perspective (Level 2)	0.090	0.089	0.048	0.042
Time (Level 2)	—	0.009	0.009	0.009
Time x Perspective (Level 2)	—	—	0.007	0.005
Residual Variance (patient perspective)	0.068	0.062	0.060	0.060
Residual Variance (therapist perspective)	0.086	0.078	0.075	0.075
*R^2^* (Level 1: patient perspective)	—	8.40%	10.87%	10.80%
*R^2^* (Level 1: therapist perspective)	—	8.98%	11.96%	11.86%
AIC	1261.207	1121.793	1088.923	1080.938

*Note:* Number of observations (Level 1) = 2797, number of patients (Level 2) = 79, number of therapists (Level 3) = 36. Age was centered on the sample mean, time was coded 1 – 24, and log transformed before the analyses. The table depicts point estimates and standard errors in parentheses. Significant values are marked in bold. ^a^0: female, 1: male: ^b^0: no comorbidity, 1: comorbid disorder; ^c^0: therapist perspective, 1: patient perspective;*p < 0.05; **p < 0.01; ***p < 0.001.

Abbreviations: AIC, Akaike information criterion; ANX, anxiety disorder, ANXvsDEP, anxiety versus depressive disorder; ANXvsHYPER, anxiety versus hyperkinetic disorder; CG, passive control group; CG‐R, control group ‐ relaxation; DEP, depressive disorder; DEPvsHYPER, depressive versus hyperkinetic disorder; HYP, hyperkinetic disorder; IG‐M, mindfulness intervention group; IG‐MvsCG, mindfulness intervention versus passive control group; IG‐MvsCG‐R, mindfulness intervention versus control group relaxation; MLM, multilevel modeling.

Next (see Model 2 in Table 4), we added the condition x perspective, condition x time, and perspective x time two‐way interactions. We found a significant interaction for group x perspective, indicating that the difference in therapeutic alliance between the two perspectives was larger in CG than in IG‐M, *b* = 0.225, *p* = 0.009. Hence, the discrepancies between patients and therapists in average alliance was lower in IG‐M. Additionally, significant group x time interactions showed that therapeutic alliance increased more strongly in IG‐M than in CG‐R, *b* = −0.072, *p* = 0.014, and more strongly in IG‐M than in CG, *b* = −0.067, *p* = 0.021. Last, the significant perspective x time interaction, *b* = −0.049, *p* = 0.016, suggested that changes in therapeutic alliance were more positive for ratings from the therapists' perspective than from the patients' perspective. Last, we added the three‐way interaction condition x time x perspective in the model (Model 3 in Table 4). This interaction is illustrated in Figure 3, which shows the change in the therapeutic alliance separately for the three groups and for the two perspectives. To uncover the nature of the interaction, this model was followed up by two models that separated the changes into patients' and therapists' perspective. Estimated marginal means of the models are reported in Table D2 (Appendix D), demonstrating from the patients' perspective an increase in IG‐M from 3.45 at S1 to 3.70 at S24, whereas CG‐R remained largely stable (from 3.46 at S1 to 3.41 at S24) and CG slightly decreased (from 3.57 at S1 to 3.42 at S24). Estimated marginal means from the therapists' perspective indicated an increase in all groups (IG‐M: 3.15 from S1 to 3.36 at S24, CG‐R: 2.92 at S1 to 3.00 at S24, CG: 2.88 at S1 to 3.11 at S24). Results showed that there were no group x time interaction effects for the ratings from the therapists' perspective, *p* > 0.291, and including group x time interaction accounted for only 7.51% of the between‐participant differences in change of therapist rated alliance over time. However, the conditional main effect of time was statistically significant, *b* = 0.065, *p* = 0.024. In sum, this pattern suggests that therapeutic alliance from the therapists' perspective increased across time, but there was no evidence that the increase in therapeutic alliance differed between the three conditions.

**Figure 3:**
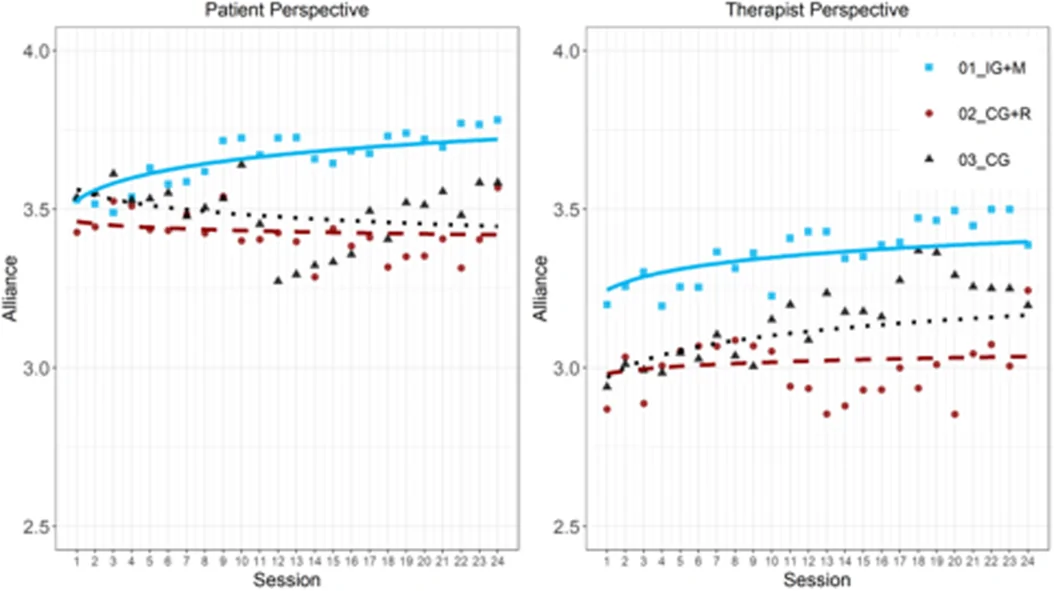
Empirical means (symbols) and estimated trajectories (lines) of therapeutic alliance, separately for IG‐M (solid blue line, ■), in CG‐R (dashed red line, •), and CG (dotted black line, ▲). Ratings from the patient perspective are depicted in the left panel, ratings from the therapist perspective in the right panel. Further information on the trajectories can be found in Table 4.

In contrast, from the patients' perspective, therapeutic alliance increased more strongly in IG‐M than in CG‐R, *b* = −0.094, *p* = 0.004, and in CG, *b* = −0.124, *p* < 0.001. Including the group x time interaction explained 27.20% of the between‐participant differences in change of patient rated alliance over time. Simple slope analyses further showed that from the patients' perspective, therapeutic alliance significantly increased across sessions in IG‐M, *b* = 0.077, *p* < 0.001, and significantly decreased in CG, *b* = −0.047, *p* = 0.048, but there was no statistically meaningful change in therapeutic alliance from the patients' perspective in CG‐R, *b* = −0.017, *p* = 0.469.

To sum up, our results were partially in line with H2 that there was a greater increase in therapeutic alliance in IG‐M than in CG‐R and CG. The statistical test family of therapeutic alliance‐related models consisted of a series of four, progressively more complex models due to the hierarchical structure of the data (main confirmatory tests). Model 0, Model 1 and Model 2 were preceding, model‐building steps, and Model 3 was the final model to answer H2 (principal test of interest; interpretation of the three‐way interaction condition x time x perspective). Additionally, due to significant interactions, two further models were set up (patients' and therapists' perspective) and followed by three simple slope analyses due to a significant interaction effect in the patients' perspective model (follow‐up analyses). In sum, within this statistical test family, six models and three simple slope analyses were conducted. No correction for multiple testing was applied, follow‐up analyses were conducted to decompose the significant interaction instead of to test independent hypotheses.

### 3.5 Response and Remission

We assessed response and remission rates based on self‐ratings of our primary outcome, the YSR 11‐18R. With regard to response, we detected 66.6% responders in IG‐M, 46.6% responders in CG‐R, and 47% responders in CG. With regard to remission, we identified 61.1% remitters in IG‐M, 40.0% remitters in CG‐R, and 41% remitters in CG. Responders and remitters did not differ across IG‐M, CG‐R, and CG (response: *χ* ^2^ (2) = 1.817, *p* = 0.40; remission: *χ* ^2^ (2) = 1.941, *p* = 0.38). The statistical test family consisted of two analyses (response analysis and remitter analysis).

## 4 Discussion

This study examined whether brief, session‐introducing mindfulness exercises in individual therapy reduce symptoms and improve the therapeutic alliance in 11‐ to 19‐year‐olds with depression, anxiety, or hyperkinetic disorder. As MBIs are often used as single mindfulness exercises during psychotherapy (Michalak et al. 2020), and potential benefits of implementing MBIs as pre‐session intervention have been outlined by former studies (e.g., Mander et al. 2019; Hunt et al. 2022), the present study aimed to implement MBIs as pre‐session intervention. To answer our research questions, we conducted an RCT with three conditions (IG‐M, CG‐R, CG) in which the effects of session‐introducing mindfulness exercises were compared to session‐introducing relaxation exercises and no session‐introducing intervention under naturalistic conditions. Please note that the results concerning therapeutic alliance have to be interpreted cautiously as they rely on higher‐order interactions, follow‐up models, and simple slope analyses.

Our analysis showed a significant symptom reduction in all groups over time. Symptoms decreased significantly more in IG‐M than in CG, but no significant differences were found between IG‐M and CG‐R or between CG‐R and CG. This is in line with former studies that already demonstrated that MBIs evoke positive effects on juveniles' symptomatology (e.g., Dunning et al. 2022; Borquist‐Conlon et al. 2019; Reangsing et al. 2021). However, the difference between mindfulness and relaxation exercises was not significant. Accordingly, the present study does not allow conclusions regarding the superiority of mindfulness interventions compared to other, more traditional relaxation exercises (Mander et al. 2019). This is also reflected by the comparable effect sizes for the pre‐post‐differences in IG‐M and CG‐R (IG‐M Cohen's *d*_*z*_: 0.73; CG‐R Cohen's *d*_*z*_: 0.63). Additionally, the observed symptom reduction cannot be confidently attributed specifically to IG‐M as results may be confounded by the more frequent use of interventions based on operant conditioning and transfer by the study therapists, demonstrated by means of the HICBI questionnaire. Furthermore, as no differences with regard to responder and remitter rates were found between the three groups (according to the guidelines of Jacobson and Truax 1991), clinical relevance of the detected group differences in favor for IG‐M is limited.

Regarding therapeutic alliance (Hypothesis 2), alliance increased over time in all groups, with a significantly greater increase in IG‐M compared to CG‐R and CG. Three‐way interaction analyses indicated that the effect of time on therapeutic alliance differed between the groups and varied by perspective (patients vs. therapists). Follow‐up analyses showed that, from therapists' perspective, alliance improved in all groups, with no significant differences between the conditions. However, from the patients' perspective, alliance increased in IG‐M, but remained unchanged in CG‐R and significantly decreased in CG. These quantitative results support the results of former qualitative studies reporting positive effects of in‐session mindfulness practice on the therapeutic alliance with adult patients (Horst et al. 2013; Konichezky et al. 2022) and extend these findings to a younger age group. The differing trajectories of alliance from therapists' and patients' perspective are consistent with former studies showing varying alliance patterns between therapists and juvenile patients (Accurso and Garland 2015; Gergov et al. 2021). Additionally, it is important to note that therapists delivered the pre‐session mindfulness exercise to the patients without taking part in it themselves. It is therefore understandable that the effects are restricted to the patients' perspective. It should be kept in mind, however, that the results regarding the therapeutic alliance rely on higher‐order interactions, follow‐up models, and simple slope analyses. These findings should therefore be interpreted very cautiously and require replication in future studies.

Many therapists already use mindfulness exercises in individual therapy without knowing their impact (Michalak et al. 2020). Until now, studies mainly relied on the application of the whole MBI with all its components such as psychoeducation, several mindfulness interventions, and group‐related factors (Mander et al. 2019, Shallcross et al. 2015). Furthermore, MBIs mostly took place in group settings (typically in schools) comprising juvenile participants with no diagnosis of a mental disorder or with sub‐clinical symptoms (e.g., Carsley et al. 2018). In the present study, a significant reduction with regard to symptomatic outcome was demonstrated in IG‐M in comparison to CG, but no significant differences were demonstrated in IG‐M in comparison to CG‐R. It remains unclear if these differences in symptomatic outcome are attributable to the different session‐introducing interventions or to other confounding variables. Additionally, no differences in response and remitter rates were detected between the three treatment conditions. Future research might focus on how mindfulness interventions, delivered as pre‐session interventions, have positive effects on existing programs of the third wave (e.g., DBT, ACT) in which mindfulness elements are already incorporated, and on how further enhance the unique benefits of mindfulness interventions over alternative session‐introducing activities by controlling potential confounding variables.

With regard to the application of therapeutic techniques, therapists applied interventions based on operant conditioning and transfer significantly more frequently in IG‐M than in the other groups. More precisely, this means that patients in IG‐M were significantly more trained in self‐monitoring using written behavioral records, more homework assignments, and more day‐ and week‐structuring activities. These types of interventions are primarily carried out by patients at home, outside of the therapy session. This may therefore, on the one hand, indicate that therapists favored patients in IG‐M by assigning them more tasks to be completed at home. Additionally, therapists might have delivered CBT in IG‐M in a way that promoted the implementation of additional therapeutic interventions between the sessions. It may also be possible that IG‐M patients, perhaps due to increased presence induced by the mindfulness intervention at the beginning of the therapy session, were more willing to carry out additional therapeutic interventions at home, which ultimately resulted in better therapeutic outcomes. It is unquestionable that homework assignments have a positive effect on therapeutic outcomes in CBT (Kazantis et al. 2010). It may thus be that the positive effects on symptomatic outcome are not only attributable to the implementation of session‐introducing mindfulness interventions, but to the engagement in homework. Furthermore, self‐monitoring using written behavioral records as well as day‐ and week‐structuring activities are also active CBT components that may positively affect therapeutic outcomes (Cuijpers et al. 2023). Hence, therapeutic techniques might be a confounding factor of our study results. As therapeutic techniques were assessed after the randomization to IG‐M, CG‐R, and CG and may thus be consequences of group allocation, accounting for these post‐randomization variables was not considered appropriate in the present work. More research is needed to disentangle the specific contribution of session‐introducing mindfulness interventions in contrast to broader CBT components on therapeutic outcomes. Further, observed group differences in CBT components should be interpreted with caution as the reliability estimates on the HICBI subscale “interventions based on operant conditioning and transfer” ranged from poor to acceptable (0.58 ≤ *ω* ≤ 0.75, for more information see Appendix B). Additionally, therapists indicated that they delivered exercising and suggestive interventions more often in IG‐M and CG‐R than in CG. The result suggests that therapists of CG followed our instruction to apply exercising and suggestive interventions (such as mindfulness and relaxation interventions) as seldom as possible to minimize the overlap with IG‐M and CG‐R. However, the interpretation of this finding is reduced due to poor reliability estimates of the according subscale across the time measurement points (HICBI subscale “exercising and suggestive interventions,” 0.12 ≤ *ω* ≤ 0.42, for more information see Appendix B). Although both HICBI subscales that showed differences between the groups had psychometric limitations with regard to their internal consistency, the HICBI is still a thoroughly validated and published diagnostic instrument (D'Errico et al. 2018). Hence, kept the HICBI's psychometric limitations in mind, results suggesting effects of therapeutic techniques based on the HICBI ratings should be taken seriously into account. It should also be noted that the HICBI was initially developed for the assessment of therapeutic techniques in adult psychotherapy, not youth psychotherapy. To date, no study investigated its feasibility in younger age groups. Therefore, the interpretability of these results is further restricted.

The results of the present study are at odds with a comparable study which investigated the effects of session‐introducing mindfulness exercises in comparison to session‐introducing relaxation exercises and no session introduction in adult outpatients (Mander et al. 2019). Mander et al. (2019) found no differences between the groups for symptomatic outcome or alliance. Several factors may explain the differences. First, the studies target different age groups (adolescents in the present study in contrast to adults in the study by Mander et al. 2019). Adolescents may respond more strongly to exercising and suggestive interventions, as it is prevalent on social media and easily accessible via apps (Creswell and Goldberg 2025), potentially lowering barriers to engagement. Furthermore, previous studies showed that effect sizes for therapeutic interventions may vary across different age groups (Cuijpers et al. 2019). Second, Mander et al. (2019) used only one mindfulness exercise (an adapted version of the ‘breathing space'; Segal et al. 2012) and PMR as relaxation exercise, while our study included three mindfulness interventions (‘mindful walking,' ‘breathing space,' ‘body scan') and three relaxation interventions (‘PMR', ‘walking relaxation', imagery journey ‘dolphin riding'), increasing intervention variety. In other words, if a patient does not respond well to one intervention, there are two other interventions that might be better fit for him. Third, Mander et al. (2019) used audio‐guided mindfulness and relaxation, whereas therapists provided direct guidance in our study. Given potential teacher effects on mindfulness outcomes (Crane 2017), therapist‐led exercises may have influenced results, as individual preferences can impact effectiveness. Additionally, direct guidance could be perceived as more personal than an audio file by the patients, potentially increasing their therapeutic engagement and commitment to the active interventions.

In sum, in addition to the fact that practicing mindfulness is more popular than ever these days (Creswell and Goldberg 2025) and that intervention effects often differ by age group (Cuijpers et al. 2019), the session‐introducing interventions used in the present study vary to those session‐introducing interventions used in Mander et al. (2019) regarding intervention variety and more personal guidance. Additionally, the pre‐session mindfulness interventions did not outperform pre‐session relaxation interventions in our study. Of note, it may also be that the varying results between the present study and Mander et al. (2019) cannot be attributed to the session‐introducing interventions, but to confounding variables such as the different use of therapeutic techniques across the treatment conditions, as already outlined above. A very recent review pointed out that mindfulness and meditation is now learned by millions of people via smartphone‐based apps (Creswell and Goldberg 2025). Future research may investigate the combination of mindfulness integrated in individual therapy with additional application‐based mindfulness training at home, thereby increasing the therapeutic dose and enhancing the effects of mindfulness interventions.

The study has some limitations. First, we lagged behind our power analyses which resulted in a sample size of 108 patients for detecting small to medium‐sized effects for treatment x time ‐ interaction on symptomatic outcome (Kalmar et al. 2022). Despite planning for 135 patients to account for a 25% dropout, recruitment challenges—such as the SARS‐CoV‐2 pandemic and parents expressing concerns about their children participating in scientific studies—prevented this. Consequently, for all model parameters including the between‐person variable treatment (main effects of the treatment and treatment x time interactions) statistical power was less than optimal which needs to be considered as a limitation of the present study. Non‐significant results may therefore not be understood as evidence of no difference, but no evidence of a difference. Future research might consider focusing on fewer conditions (e.g., only IG‐M and CG‐R) and increase the sample size per condition to increase power.

Nevertheless, as our study is one of the few targeting mindfulness in individual therapy for children and adolescents, these findings are of high scientific importance. Furthermore, a sample size of 90 adolescent patients is comparable to the sample sizes of other RCTs conducted in the field of CBT with youth (e.g., Dunning et al. 2022; Zhou et al. 2020). Second, the study's naturalistic design ensured high external validity but may have affected internal validity. To prevent therapy duration differences, interventions were integrated into sessions, limiting their impact to the first 5–10 min (Bell et al. 2013; Cuijpers et al. 2019). Third, we log‐transformed our measurement time points before we added it our statistical models. Whereas the coded time values represent the accumulation of therapy sessions, this approach does not reflect variability in the actual intervals between assessments. Additionally, log‐transforming of time assumes the dose‐response model which postulates that the degree of symptomatic improvement is related to the dose (i.e., number of therapy sessions), with the effect of dose getting weaker over time (Howard et al. 1986). Although this is generally in line with previous work (Robinson et al. 2020a), it has also been questioned as the relationship of dose and response may result from combining different patients' subgroups (such as rapid responders and slow responders). Other possibilities of time modeling such as quadratic, cubic, piece‐wise linear, and unstructured approaches (Crits‐Christoph et al. 2023) could be considered as well, though these models require more parameters than our chosen more economical approach. Fourth, we were not able to assess reliability of adherence as adherence was only rated by one rater. Fifth, with regard to the verification of diagnostic assessments, diagnostic evaluation of the diagnoses comprised only the assessment of the primary diagnosis, not the comorbid diagnoses. The reliability of symptom‐level or comorbid diagnostic information could not be established. As 40% of the patients in our sample exhibited comorbid diagnoses, this missing diagnostic evaluation might affect the covariate adjustment in our statistical analyses. Results comprising the comorbid diagnoses must therefore be interpreted with caution. Sixth, the results of the present study may be confounded by other variables, particularly by the use of therapeutic techniques. We detected that therapists in the IG‐M used more interventions based on operant conditioning and transfer than therapists in the CG‐R and CG. Results in favor of the IG‐M may thus not only be attributable to the implementation of session‐introducing mindfulness interventions, but to the more frequent use of specific CBT methods. Internal validity of the present study is therefore limited. Seventh, the results regarding the therapeutic alliance rely on higher‐order interactions, follow‐up models, and simple slope analyses and thus require replication in future studies.

Our study largely follows the recommendations by Michalak et al. (2019) for a responsible application of mindfulness in individual therapy. As the mindfulness exercises were part of an RCT, the mindfulness exercises were not tailored to the individual patients and their case conceptualizations, as recommended by Michalak et al. (2019). Nevertheless, we ensured that the mindfulness exercises are likely to have positive effects on the symptomatology of the mental disorders (namely depression, anxiety, and hyperkinetic disorder) in our sample, as confirmed by meta‐analyses (Borquist‐Conlon et al. 2019; Lee et al. 2022; Reangsing et al. 2021). Additionally, patients had the option to quit study participation if they experienced adverse effects due to the exercises.

Our study provides several clinical implications. We detected significant effects of single, session‐introducing mindfulness interventions in comparison to no session‐introducing interventions on therapeutic outcomes, but no significant differences between session‐introducing mindfulness interventions and session‐introducing relaxation interventions. It remained unclear whether treatment effects could be attributed to mindfulness or confounding variables (e.g., therapeutic techniques). Using a variety of exercising and suggestive interventions is recommended to maintain engagement in youth. As the present study took place in a naturalistic outpatient setting including patients with different main diagnoses and additional comorbid disorders, single exercising and suggestive interventions seem to be suited for a broad spectrum of psychopathology during adolescence. Results are preliminary as no differences with regard to response and remission rates were detected between the three treatment conditions and results were probably confounded by therapeutic techniques, particularly by the application of interventions based on operant conditioning and transfer. With regard to therapeutic alliance, session‐introducing mindfulness interventions have predominantly positive effects on patients' side, but not on therapists' side. However, these results of therapeutic alliance have to be interpreted very cautiously as they were based on higher‐order interactions, follow‐up models, and simple slope analyses and thus require replication in future studies.

In sum, mindfulness is still a hype in psychology (van Dam et al. 2018; Creswell and Goldberg 2025) and many clinicians apply mindfulness in individual psychotherapy (Michalak et al. 2020). Hence, empirical studies investigating mindfulness interventions are of high clinical relevance. The specific effects of mindfulness in the current study remained unclear, as specific CBT techniques, such as interventions based on operant conditioning and transfer, might have confounded the effects of session‐introducing mindfulness. These potentially confounding variables constitute the central limitation in interpreting the study's primary outcome. Additionally, results concerning therapeutic alliance (significant increase over time from the therapists' perspective without group differences, significant increase in IG‐M, significant decrease in CG, and no meaningful change in CG‐R over time from the patients' perspective) rely on higher‐order interactions, follow‐up models, and simple slope analyses. Non‐significant results may therefore not be understood as evidence of no difference, but no evidence of a difference.

Future studies should very carefully address this aspect by using multi‐perspective therapeutic techniques assessment (e.g., observer, patient, and therapist ratings) and by experimentally manipulate the use of therapeutic techniques in combination with mindfulness (e.g., mindfulness condition with vs. without operant conditioning and transfer techniques). If multiple data related to mindfulness and therapeutic techniques are collected in individual CBT, this might eventually shed light on the potentials and limitations of mindfulness in this area and thereby provide empirically based guidelines for practitioners using mindfulness in individual therapy.

## Ethics Statement

The local ethics committee approved the study protocol in accordance with the Helsinki Declaration.

## Consent

Informed consent was obtained from all patients before participation in the study.

## Conflicts of Interest

T.H. and J.M. receive royalties from mindfulness books they have authored. T.H. and J.M. receive stipends from workshops on mindfulness. The other authors declare no conflicts of interest.

## Clinical Trial Registration

This trial was registered at ClinicalTrials. gov (NCT04034576).

## Permission to Reproduce Material From Other Sources

Our manuscript does not obtain material from other sources.

## Supporting information


Supporting File 1

Supporting File 2

Supporting File 3

Supporting File 4

## Data Availability

Data are available on request from the authors.

## Footnotes

1 In the study protocol, we also stipulated a third hypothesis that the mindfulness session introduction would moderate the association between therapeutic alliance and symptoms. However, as the resulting sample was most likely underpowered to detect the postulated interaction effect between two person‐level variables (condition and average therapeutic alliance), we decided to not explicitly include the results on this hypothesis in the present manuscript. In the interest of full transparency, the results of these analyses are reported in Appendix A of this work. We note, however, that the results reported in Appendix A need to be interpreted particularly carefully given the limited statistical power of these analyses.

2 We further explored potential differences between CG‐R and CG by re‐estimation Model 2 with two dummies contrasting CG and IG‐M, and CG and CG‐R, respectively. The CGvsCG‐R x time interaction yielded no evidence for a difference between CG and CG‐R in the amount of symptom change over time, *b* = −5.137, *p* = 0.213.
