# Supplementary material for: The Impact of Mindfulness as a Session‐Introduction on Symptoms and Alliance in Individual Cognitive‐Behavioral Therapy With Children and Adolescents: A Randomized Controlled Trial
Source: J Clin Psychol. 2026 Aug 29;82(12):2007–25. doi: 10.1002/jclp.70216 (PMC13733648; doi:10.1002/jclp.70216)
Supplement: Supplementary file 1 [file JCLP-82-2007-s001.docx]

**Appendix B. Treatment integrity**

**Measures**

Adherence of the patients and of the therapists to the interventions was assessed via self-designed questionnaires and via video-based observer ratings. The questionnaires consist of five (patient) respective three items (therapist) rated on a five-step scale with 0 (does not apply) to 4 (applies exactly). Three items of the questionnaire are parallelized between patients and therapists, and ask them whether the intervention was understandable, or whether the intervention could be integrated into the therapy session without circumstances. Patients of the study were additionally asked to complete two specific questions concerning the execution of the intervention. Furthermore, therapists were asked whether they implemented a session-introducing intervention and if so, which one. This was used to check whether the correct intervention was applied.

Video-based observer ratings of adherence (six items in total) were conducted to give information whether the interventions were executed in an adequate manner. Additionally, observers assessed whether therapists did not apply session-introducing interventions in CG.

For the assessment of in-session therapeutic presence of both patients and therapists, an adapted version (Mander et al., 2019) of the Therapist Presence Inventory (TPI; Geller et al., 2010) was applied. The original version of the TPI (Geller et al., 2010) shows an internal consistency of α = .75, demonstrates concurrent validity to a relationship measure and predictive validity to an alliance measure (Geller et al., 2010). Both versions (therapist, patient) of the TPI consist of six items, and are rated on a five-step scale from 0 (does not apply at all) to 4 (applies exactly). Items of both versions are parallelized to each other. The questionnaire is originally developed for adult patients and is applied to children and adolescents in a linguistic adapted form in the current study. The internal consistencies in the current sample were ω = .82 (S3), ω = .86 (S10), ω = .87 (S17), and ω = .86 (S24) for the therapist ratings. For the patient ratings, the internal consistencies in the present sample were ω = .78 (S3), ω = .87 (S10), ω = .92 (S17), and ω = .84 (S24).

To assess the perceived quality of the session-introducing intervention, the Practice Quality-Mindfulness (PQ-M; Del Re et al., 2013) was applied to the patients of the study. The PQ-M consists of six items, is rated on a 5-step scale from 0 (does not apply at all) to 4 (applies exactly), and two subscales (present-moment attention: items 1 to 3; receptive attention: items 4 to 6; Del Re et al., 2013). The measure shows moderate to large internal consistencies with .66 ≤ α ≤ .87 in previous studies (Del Re et al., 2013). Convergent validity is given by significant positive correlations to other mindfulness measures (Del Re et al., 2013). The questionnaire is originally developed for adult patients and is applied to children and adolescents in a linguistic adapted form in the current study. In the present sample, the internal consistency for the subscale present-moment awareness were ω = .64 (S3), ω = .84 (S10), ω = .81 (S17), and ω = .73 (S24). For the subscale receptive attention, the internal consistencies in the present sample were ω = .66 (S3), ω = .70 (S10), ω = .76 (S17), and ω = .74 (S24).

Therapeutic competence of the therapists was assessed by means of the Cognitive Behaviour Therapy Scale for Children and Young People (CBTS-CYP; Stallard et al., 2014), an adaptation of the Cognitive Therapy Scale – Revised (CTS-R; Blackburn et al., 2001) for youth. The questionnaire consists of 15 items and is rated by independent observers on a 7-step scale from 0 (deficient) to 6 (excellent), in line with the rating system of the German version of the CTS-R (Weck et al., 2010). Interrater reliabilities in the present study, measured by the intraclass coefficient (ICC), ranged between .35 ≤ ICC ≤ .88.

Allegiance effects of patients and therapists concerning the offered treatment were assessed via the Credibility/Expectancy Questionnaire (CEQ; Devilly & Borkovec, 2000). The CEQ is available in a patient and therapist version and consists of six items, rated either on a 9-step scale (items 1, 2, 3, 5) from 1 (not useful at all) to 9 (very useful) or from 0% to 100% (items 4, 6) (Devilly & Borkovec, 2000). The questionnaire comprises the two subscales ‘treatment expectancy’ and ‘credibility’ and has shown good internal consistencies between .79 ≤ α ≤ .90 in previous work (Cevilly & Borkovec, 2000). The expectancy factor is outcome predictive (Cevilly & Borkovec, 2000). The questionnaire is originally developed for adult patients and is applied to children and adolescents in a linguistic adapted form in the current study. Internal consistencies in the present study from the therapist perspective were for the two subscales credibility / expectancy ω = .77 / ω = .90 (S3), ω = .71 / ω = .92 (S10), ω = .82 / ω = .95 (S17), and ω = .81 / ω = .95 (S24). For the patient perspective ratings, the internal consistencies for the two subscales credibility / expectancy were ω = .84 / ω = .88 (S3), ω = .81 / ω = .91 (S10), ω = .82 / ω = .91 (S17), and ω = .86 / ω = .95 (S24).

To gain insight in the applied therapeutic techniques of the therapists, the ‘Heidelberg Inventory of Cognitive-Behavioral Interventions’ (HICBI; (D’Errico et al., 2018) was applied. The HICBI is rated on a five-step scale from 0 (not applied) to 4 (applied very often) by the therapists themselves. It consists of the five subscales ‘alliance’, ‘cognitive interventions’, ‘interventions based on operant conditioning and transfer’, ‘exposition’, and ‘exercising and suggestive interventions’. In prior studies, internal consistencies of the subscales ranged between .59 ≤ α ≤ .82, convergent validity, measured by the association of the HICBI subscales to a measure of general change, ranged between -.01 ≤ *r* ≤ .26 (D’Errico et al., 2018). Internal consistencies at the four measurement occasions in the present work ranged between .72 ≤ ω ≤ .86 (alliance), .47 ≤ ω ≤ .77 (cognitive interventions), .58 ≤ ω ≤ .75 (interventions based on operant conditioning and transfer), and .12 ≤ ω ≤ .42 (exercising and suggestive interventions

For the subscale exposition consisting of two items, reliability was estimated as the correlation between the two items, which ranged from *r* = .19 (S17) to *r* = .54 (S24).

**Table Appendix B. Results of Treatment Integrity**

| **Adherence to the Interventions, Questionnaire** | | | **Perspective** | | |  | |  |
| --- | --- | --- | --- | --- | --- | --- | --- | --- |
|  | | | **Therapist** | **Patient** | **Observer** |  | |  |
|  | **Mindfulness Condition, M *(SD)*** | | 3.00 *(0.56)* | 3.38 *(0.43)* | 3.63 *(0.41)* |  | |  |
|  | **Relaxation Condition, M *(SD)*** | | 3.16 *(0.71)* | 2.76 *(0.42)* | 3.44 *(0.61)* |  | |  |
|  | | |  |  |  |  | |  |
| **Adherence to the Interventions, Video Ratings** | | | | | | | |  |
|  | **All interventions matched the assigned condition** | | applicable (except two times) |  |  |  | |  |
|  | **Exceptions** | | | Accidentally implementation of the mindfulness exercise in the CG-R | | | |  |
|  | **Non-implementation of the intervention in the IG-M** | | Three times |  |  |  | |  |
|  | **Reasons for non-implementation of the intervention in the IG-M** | | | No stable internet connection during video-based therapy; Forgotten; Omitted due to a concurrent CBT intervention (exposition) | | | |  |
|  | **Non-implementation of the intervention in the CG-R** | | Five times |  | | | |  |
|  | **Reasons for non-implementation of the intervention in the CG-R** | | | No stable internet connection during video-based therapy; Forgotten; Patients refused to take part in the intervention | | | |  |
|  | | |  |  |  |  | |  |
| **Practice Quality-Mindfulness** | | |  | **Patient** |  |  | |  |
|  | **Mindfulness Condition, M *(SD)*** | |  | 2.74 *(0.35)* |  |  | |  |
|  | | |  |  |  |  | |  |
| **Therapist Presence Inventory** | | |  |  |  |  | |  |
|  | | | **Therapists’ self-rating** | **Therapists’ rating of their patients** | **Patients’ self-rating** | **Patients’ rating of their therapists** | |  |
|  | **Mindfulness condition, M *(SD)*** | | 3.34 *(0.38)* | 3.12 *(0.44)* | 2.96 *(0.62)* | 3.76 *(0.30)* | |  |
|  | | |  |  |  |  | |  |
| **Allegiance** | | |  |  |  |  |  | |
|  | **CEQ - Therapist** | | **IG-M** | **CG-R** | **CG** | **F_(2,74)_** | ***p*** | |
|  |  | Treatment expectancy, M *(SD)* | 5.94 *(1.2)* | 5.50 *(1.27)* | 6.03 *(1.32)* | 1.28 | .29 | |
|  |  | Credibility, M *(SD)* | 7.18 *(0.94)* | 6.87 *(1.22)* | 6.94 *(1.08)* | 0.56 | .57 | |
|  |  |  |  |  |  |  |  | |
|  | **CEQ - Patient** | | **IG-M** | **CG-R** | **CG** | **F_(2, 71)_** | ***p*** | |
|  |  | Treatment expectancy, M *(SD)* | 5.91 *(1.75)* | 5.48 *(1.67)* | 6.53 *(1.61)* | 2.80 | .07 | |
|  |  | Credibility, M *(SD)* | 7.11 *(1.39)* | 6.77 *(1.30)* | 7.24 *(1.27)* | 0.82 | .44 | |
|  |  |  |  |  |  |  |  | |
| **Therapists’ Competence, Observer Rating** | | | |  |  |  |  | |
|  | **CBTS-CYP** | | **IG-M** | **CG-R** | **CG** | **F_(2,33)_** | ***p*** | |
|  |  | Total Score, M *(SD)* | 3.86 *(0.29)* | 3.89 *(0.69)* | 3.90 *(0.52)* | 0.02 | .98 | |
|  |  | |  |  |  |  |  | |
| **Applied CBT Techniques** | | |  |  |  |  |  | |
|  | **HICBI** | | **IG-M** | **CG-R** | **CG** | **F_(2, 74)_** | ***p*** | |
|  |  | Alliance, M *(SD)* | 1.08 *(0.72)* | 1.14 *(0.75)* | 0.96 *(0.74)* | 0.41 | .67 | |
|  |  | Cognitive Interventions, M *(SD)* | 0.93 *(0.50)* | 0.65 *(0.43)* | 0.70 *(0.39)* | 2.92 | .06 | |
|  |  | Exposition, M *(SD)* | 0.35 *(0.59)* | 0.29 *(0.41)* | 0.28 *(0.53)* | 0.12 | .89 | |
|  |  | Interventions Based on Operant Conditions and Transfer, M *(SD)* | 1.94 *(0.75)* | 1.14 *(0.14)* | 1.34 *(0.80)* | 7.73 | **<.001^a^** | |
|  |  | Exercising and Suggestive Interventions, M *(SD)* | 1.14 *(0.41)* | 1.12 *(0.48)* | 0.42 *(0.45)* | 33.19 | **<.001^b^** | |

^a^: more often applied in the IG-M in comparison to CG-R and CG, Tukey post-hoc *p* <.001.

^b^: more often applied in the IG-M and CG-R in comparison to CG, Tukey post-hoc *p* <.001.

Abbrevations: CBT = Cognitive-Behavioral Therapy; CBTS-CYP = Cognitive Behaviour Therapy Scale for Children and

Young People; CEQ = Credibility/Expectancy Questionnaire; CG = Control Group; CG-R = Active Control Group – Relaxation;

HICBI = Heidelberg Inventory of Cognitive-Behavioral Interventions;IG-M = Index Group – Mindfulness; M = mean;

SD = Standard Deviation.
