# Supplementary material for: The Impact of Mindfulness as a Session‐Introduction on Symptoms and Alliance in Individual Cognitive‐Behavioral Therapy With Children and Adolescents: A Randomized Controlled Trial
Source: J Clin Psychol. 2026 Aug 29;82(12):2007–25. doi: 10.1002/jclp.70216 (PMC13733648; doi:10.1002/jclp.70216)
Supplement: Supplementary file 2 [file JCLP-82-2007-s002.docx]

**Appendix C. Interventions**

Mindfulness interventions:

**Breathing space**

Your posture in the chair is comfortable, you are sitting upright with your feet flat on the floor. Your hands are resting loosely on your legs. If it is comfortable for you, you can close your eyes. But you can also keep your eyes open and look at a point on the floor. [5-second pause].

Take a deep breath in and out. Now continue breathing calmly [10-second pause].

Then ask yourself: What do I notice...in my thoughts...in my feelings...and in my body? [15-second pause]

Take note of everything around you, whether pleasant or unpleasant [20-second pause].

Then gently bring your full attention to your breathing, each inhalation and exhalation as they follow one another.

By focusing on your breathing, you will become more and more aware of the here and now [15-second pause]. You may notice how your stomach rises when you inhale and falls again when you exhale. [15-second pause].

If your thoughts wander, that's perfectly fine, it just happens. When your thoughts have wandered, simply recognize this and gently bring your attention back to your breathing [15-second pause].

And now expand your attention and simply notice the sensations in the rest of your body. The sensations you experience are just fine the way they are [10-second pause]. You may notice your posture or your facial expression [10-second pause]. Open yourself to all the sensations that are unfolding, be they thoughts, feelings or bodily sensations [20-second pause].

Then, when you are ready, let go of these sensations. Slowly open your eyes with the intention of bringing this attention to the current moment and the rest of the day [15-second pause].

Talk time: 130 seconds, Pause 150 seconds, Total: 280 seconds

**Body scan**

Your posture in the chair is comfortable, you are sitting upright with your feet flat on the floor. Your hands are resting loosely on your legs. If it is comfortable for you, you can close your eyes. But you can also keep your eyes open and look at a point on the floor. [5-second pause].

Pay attention to your belly as you breathe. Notice how your belly rises and falls with each breath [15-second pause].

If your thoughts wander, that's perfectly fine, it just happens. When your thoughts have wandered, simply recognize this and gently bring your attention back to your breathing [15-second pause].

Then direct your attention to the right arm. Observe how your awareness moves down the right arm until it reaches the hand. Shift your attention to your individual fingers as they rest quietly on the leg together with your hand. Do your best to be aware of your sensations in the here and now [15-second pause].

Now focus your attention on your left arm. Let your attention slowly move down your left arm until it reaches your hand. Observe how it moves to each individual finger. Again, the left hand and fingers are resting quietly on the leg.

Whether you experience pleasant or unpleasant sensations, try to simply notice and accept them [15-second pause].

Now direct your attention to your head. Notice the back of your neck, the area around your face with your eyes, nose, mouth and ears. Feel the air flowing in and out of your nose. While you do this, stay as much as possible in the here and now. Then let your attention move to your scalp and stay there for a moment [15-second pause].

Now try to focus your attention on your back, shoulders and stomach. Observe how the back, shoulders and abdomen are gently moving with the breathing [5-second pause]. It does not matter whether the sensations felt are pleasant or unpleasant. Just accept them as they are. Allow yourself to linger on the back, shoulders and abdomen for a few more breaths [15-second pause].

Now shift your attention to your right leg. Let your attention slowly descend down your right leg until you reach your right foot. Feel how the foot rests on the floor. Now shift your attention to each individual toe. You feel the toes touching the floor. Whatever you perceive is okay. Try to simply accept these sensations as best you can [15-second pause].

Now shift your attention to your left leg. Again, slowly move your attention down until you reach your left foot. Observe how the foot rests on the floor. Stay in the here and now as much as possible. Then move on to the left toes. Again, feel how each individual toe touches the ground [15-second pause].

After you have scanned your entire body in this way, spend some time becoming aware of your entire body. Your breath flows freely in and out of your body [15-second pause].

Then, when you are ready, let go of these sensations. Slowly open your eyes with the intention of bringing this attention to the current moment and the rest of the day [15-second pause].

Talk time: 295 seconds, Pause 155 seconds, Total: 450 seconds

**Mindful walking**

Your posture in the chair is comfortable, you are sitting upright with your feet flat on the floor. Your hands are resting lightly on your legs [5-second pause].

We can discover and practise mindfulness while walking. We can walk to experience the act of walking and not to achieve any particular goal. We can use the sensations of walking to be more in the here and now [5-second pause].

We begin this exercise by slowly standing up and remaining in place. Notice the feeling of the ground beneath your feet. If you notice any other sensations, that's fine. Simply notice what is there [5-second pause].

Feel grounded in the present. Be aware of the intention to move. We will now take a step with each foot in slow motion. Now start moving. Lift one foot very slowly, move the foot forward, place the heel on the floor, then roll the foot forward to the tiptoes. You will notice how the other foot moves along as if by itself and follows suit. Now both feet are next to each other again. Feel the body resting and connected to the ground. Also notice your thoughts, which arise together with every sensation [5-second pause].

Then slowly lift the other foot, move the foot forward, place the heel on the floor, and roll the foot forward on the floor to the tiptoes. How does it feel when you roll the foot forward on the floor and then lift it up again to take the next step? Whether it's pleasant or unpleasant sensations, try to accept them as they are. Stay in the here and now as much as possible [5-second pause]. If you notice that your thoughts have wandered, gently bring your attention back to your physical sensations [5-second pause].

Walk up and down in this way, being as aware as you can of the physical sensations in your feet and the ground. Look ahead. At the beginning, walk at a slower pace than normal so that you can fully perceive the sensations as you walk [15-second pause].

You can also try walking faster, up to and beyond your normal walking pace. Sometimes it can be helpful to start by walking faster and then naturally slowing down to help yourself calm down as well [15-second pause].

Then, when you are ready, let go of these sensations. Finish by intending to bring this attention to the current moment and the rest of the day [15 seconds].

Talk time: 185 seconds, Pause 70 seconds, Total: 255 seconds

Relaxation interventions:

**Walking relaxation**

Your posture in the chair is comfortable, you are sitting upright with your feet flat on the floor. Your hands are resting lightly on your legs [5-second pause].

Start clapping your hands in a rhythm that you find relaxing. By clapping the beat, you will notice how a pleasant feeling of relaxation can slowly spread through you [15-second pause].

When you feel you have internalized the beat, stand up and try to walk to the beat. Keep clapping your hands. By clapping and walking at the same time, your feeling of relaxation will increase even further. You will notice how the tension you have built up over the course of the day can slowly be released [15-second pause].

Try changing the beat. Clap your hands a little faster and adjust your pace to the slightly faster beat [15-second pause].

Now try clapping your hands a little slower. The slower clapping will also make your walking slower. As you do this, you will feel yourself becoming more and more relaxed and a pleasant feeling will spread through you [15-second pause].

Now decide for yourself how fast or slow you want to clap and walk. Try out what feels relaxing for you and what doesn't feel so relaxing on you. Switch between different clapping speeds as often as you like [20-second pause].

Now clap again at the speed at which you felt the most relaxation after the different clapping speeds. As you do this, continue to notice how the relaxation can spread throughout your body. Everything becomes very pleasant and relaxed [20-second pause].

Then, when you are ready, let go of these sensations. End the exercise with the intention of bringing this relaxation into the current moment and the rest of the day [15-second pause].

Talk time: 135 seconds, Pause: 120 seconds, Total: 255 seconds

**Dolphin riding**

Your posture in the chair is comfortable, you are sitting upright with your feet flat on the floor. Your hands are resting loosely on your legs. If it is comfortable for you, you can close your eyes. But you can also keep your eyes open and look at a point on the floor. [5-second pause].

Imagine traveling the world's oceans in an underwater boat. You see many beautiful things underwater [5-second pause].

During one of your underwater excursions, you want to explore the area a bit. So you slide out of the exit hatch of your underwater boat into the pleasantly warm water. You touch the fine, soft sand of the seabed. There is said to be a pod of dolphins nearby, which you are very much looking forward to seeing. You swim past many colorful swarms of fish [5-second pause].

On your way to the pod of dolphins, you feel completely calm and safe. You feel the warm water on your body. You say to yourself: *My body is very warm! My body is very warm!* [5-second pause]

You see the dolphins frolicking in a nearby part of the sea. You sit down on a large rock and watch the agile dolphins. You could watch them for hours as they move their flukes back and forth and glide their slender bodies through the water. You marvel at how skillfully the dolphins swim around in the water, jump out of the water and shoot back into the water like arrows [5-second pause].

After a while, a dolphin comes swimming close to you. It is very tame, swimming around you and it seems as if it wants to greet you. As you sit on the rock watching, you notice the warmth of the water surrounding the center of your body, around your belly, and you say to yourself: *My belly is very warm! My belly is very warm!* [5-second pause]

You gently caress the dorsal fin of the dolphin. After you feel that the dolphin trusts you, you hold on to its dorsal fin and it pulls you through the water. You glide weightlessly and effortlessly through the magnificent, bright, warm water. You feel your body getting pleasantly warm again. You say to yourself: *My body is very warm! My body is very warm!* [5-second pause]

After a while, you decide to leave the dolphin and the pod and return to your underwater boat. As a farewell, you caress the dolphin again and he lovingly nudges you with his snout. You glide slowly and safely back to the underwater boat. Soon it appears in the clear water in front of you. The sun makes it shine, and the colors are even more intense than on land. You have arrived and enter through the hatch. You feel calm and content [10-second pause].

Then, when you are ready, let go of these sensations. Slowly open your eyes with the intention of bringing this relaxation into the present moment and the rest of the day [15-second pause].

Talk time:220 seconds, Pause: 60 seconds, Total: 280 seconds

**Progressive muscle relaxation**

Your posture in the chair is comfortable, you are sitting upright with your feet flat on the floor. Your hands are resting loosely on your legs. If it is comfortable for you, you can close your eyes. But you can also keep your eyes open and look at a point on the floor. [5-second pause].

Take a few deep breaths in and out [5-second pause].

Now continue breathing calmly.

We will focus on certain muscle groups in a moment. When I say “now,” tense your muscles. After a few seconds, you can relax them again when I say “and let go” [5-second pause].

Now focus on your right arm. Bend your right arm, clench your hand into a fist and tense as many muscles of your upper and forearm as possible. Now [5-second pause].

And let go. Let your arm drop gently, unclench your fist and relax all the muscles. Feel the relaxed feeling in your whole arm. Pay close attention to the easing of tension and the emerging relaxation [10-second pause].

Now focus on your left arm. Bend your left arm, clench your hand into a fist and tense all the muscles of your upper and lower arm as much as possible. Now [5-second pause].

And let go. Let your arm drop gently, unclench your fist and relax all the muscles. Feel the relaxed feeling in your whole arm. Again, concentrate fully on the easing of the tension and the onset of relaxation [10-second pause].

We now move to the face. Squint your eyes and clench your teeth. Make a grimace with your whole face. Now [5-second pause].

And let go. Let your face become completely relaxed and smooth. Pay attention to how it feels when the muscles in your whole face relax more and more... Let go completely... with each exhalation the relaxation becomes deeper and deeper [10-second pause].

Now we move to the back, shoulders and stomach. Take a deep breath, pull your shoulders back and draw in your stomach. Now [5-second pause].

And let go. Exhale, relax your back, shoulders and stomach completely. Continue breathing calmly. Pay attention to the difference between tensing and relaxing... Let go of all your muscles... Concentrate on the relaxed feeling in your entire upper body, which will help you to relax more and more [10-second pause].

And now to your right leg. Lift your right leg slightly off the seat, pull your toes towards you and turn your foot slightly inwards. Now [5-second pause].

And let go. Put your leg back down in a comfortable position and relax all your muscles. Notice the difference between tensing and relaxing. Let go completely. Feel how relaxed your whole leg feels. Everything is very pleasant and relaxed [10-second pause].

And now to your left leg. Lift your left leg slightly off the seat, pull the tips of your toes towards you and turn your foot slightly inwards. Now [5-second pause].

And let go. Put your leg back down in a comfortable position and relax all your muscles. Notice the difference between tensing and relaxing. Let go completely... Feel how the relaxation feels in your whole leg. Again, everything is very pleasant and relaxed [10-second break].

Finally, please tense all the muscle groups that we have just gone through together: the arms, the face, the back, the shoulders, the belly and the legs. Now [5-second pause].

And let go. Surrender to the feeling of relaxation for a few breaths, so that it can spread throughout your entire body. The relaxation expands more and more and becomes deeper and deeper [10-second pause].

Then, when you are ready, let go of these sensations. Slowly open your eyes with the intention of bringing this relaxation into the present moment and the rest of the day [15-second pause].

Talk time: 315 seconds, Pause: 135 seconds, Total: 450 seconds
