# Supplementary material for: The Impact of Mindfulness as a Session‐Introduction on Symptoms and Alliance in Individual Cognitive‐Behavioral Therapy With Children and Adolescents: A Randomized Controlled Trial
Source: J Clin Psychol. 2026 Aug 29;82(12):2007–25. doi: 10.1002/jclp.70216 (PMC13733648; doi:10.1002/jclp.70216)
Supplement: Supplementary file 3 [file JCLP-82-2007-s003.docx]

**Appendix D. Estimated Marginal Means**

Table D1. Estimated Marginal Means - Symptoms (Model 2, Table 3)

| Session | Estimated marginal mean IG-M | Estimated marginal mean CG-R | Estimated marginal CG |
| --- | --- | --- | --- |
| Pre | 57.6 [47.5, 67.7] | 59.9 [50.0, 69.7] | 51.0 [41.1, 60.6] |
| S3 | 49.7 [40.1, 59.2] | 56.8 [47.3, 66.2] | 51.4 [42.0, 60.8] |
| S10 | 45.0 [35.0, 55.1] | 54.9 [44.9, 65.0] | 51.7 [41.6, 61.7] |
| S17 | 41.8 [31.1, 52.4] | 53.6 [42.9, 64.4] | 51.8 [41.1, 62.6] |
| S24 | 39.2 [27.9, 50.5] | 52.6 [41.1, 64.1] | 52.0 [40.5, 63.3] |

*Note*. Table depicts the model implied estimated marginal means [95% confidence interval in brackets] for the primary outcome symptoms at pre and at sessions S3, S10, S17 and S24.

Table D2. Estimated Marginal Means – Therapeutic Alliance

|  | Session | Estimated marginal mean IG-M | Estimated marginal mean CG-R | Estimated marginal CG |
| --- | --- | --- | --- | --- |
| Patient Perspective | S1 | 3.45 [3.30, 3.61] | 3.46 [3.31, 3.61] | 3.57 [3.41, 3.73] |
|  | S3 | 3.54 [3.39, 3.69] | 3.44 [3.30, 3.59] | 3.52 [3.36, 3.67] |
|  | S10 | 3.63 [3.48, 3.79] | 3.42 [3.27, 3.57] | 3.46 [3.30, 3.62] |
|  | S17 | 3.67 [3.51, 3.84] | 3.41 [3.25, 3.58] | 3.44 [3.27, 3.61] |
|  | S24 | 3.70 [3.53, 3.87] | 3.41 [3.23, 3.58] | 3.42 [3.24, 3.60] |
| Therapist perspective | S1 | 3.15 [2.97, 3.33] | 2.92 [2.75, 3.10] | 2.88 [2.70, 3.06] |
|  | S3 | 3.22 [3.07, 3.38] | 2.95 [2.80, 3.10] | 2.96 [2.81, 3.12] |
|  | S10 | 3.30 [3.14, 3.46] | 2.98 [2.82, 3.14] | 3.05 [2.89, 3.21] |
|  | S17 | 3.33 [3.16, 3.51] | 2.99 [2.82, 3.16] | 3.09 [2.91, 3.26] |
|  | S24 | 3.36 [3.17, 3.54] | 3.00 [2.82, 3.18] | 3.11 [2.93, 3.30] |

*Note*. Table depicts the model implied estimated marginal means [95% confidence interval in brackets] for the secondary outcome therapeutic alliance at sessions S1, S3, S10, S17 and S24. Results are reported separately for alliance rated from the patients’ perspective and from the therapists’ perspective.
