# Supplementary material for: The Impact of Mindfulness as a Session‐Introduction on Symptoms and Alliance in Individual Cognitive‐Behavioral Therapy With Children and Adolescents: A Randomized Controlled Trial
Source: J Clin Psychol. 2026 Aug 29;82(12):2007–25. doi: 10.1002/jclp.70216 (PMC13733648; doi:10.1002/jclp.70216)
Supplement: Supplementary file 4 [file JCLP-82-2007-s004.docx]

**Appendix A**

**Hypothesis 3: Is the association between therapeutic alliance and outcome moderated by mindfulness?**

Former studies have shown significant associations between therapeutic alliance and treatment outcome (e.g. Karver et al., 2018). Therapeutic alliance may be enhanced by MBIs, as the implementation MBIs fosters empathy and self-compassion (Kristeller & Johnson et al., 2005). Therefore, it may be possible that the implementation of MBIs has an impact on the association between therapeutic alliance and symptomatic outcome. However, no study has investigated the moderating effect of MBIs on the association between therapeutic alliance and symptomatic outcome in children and adolescents yet. We hypothesized that the relationship between therapeutic alliance and symptomatic outcome over time is moderated by IG-M (Hypothesis 3).

To test this hypothesis, we first centered the mean values of the alliances on their respective sample means. We then included these centered mean values of therapeutic alliance over the 24 sessions for the therapist and the patient in the previously described model for the prediction of psychopathological outcomes (i.e., prediction of psychopathological outcomes by the main effects of condition, [logarithmized] time, age, gender, comorbidity, and disorder group, and condition x time two-way interaction [Model 2 in Table 4]). Adding the mean values did not significantly improve model fit, χ²(2) = 1.17, *p* = .557, suggesting that (average) therapeutic alliance was not linked to (average) psychopathological symptomatology. Model results were in line with this assumption: There were no significant effects of mean alliance patient and mean alliance therapist on psychopathological symptomatology, *p* > .380. Additionally, adding the two-way interactions mean alliance patient x condition, mean alliance therapist x condition, mean alliance patient x time, and mean alliance therapist x time did not improve the model fit, χ²(6) = 5.629, *p* = .466. We found a significant two-way interaction for IG-MvsCG x time, *b* = 12.183, *p* = 0.004, none of the other two-way interactions were significant, *p* > .102. Last, adding the three-way interactions did again not improve model fit, χ²(4) = 1.51, *p* = .825, and we found no significant three-way interactions, *p* > .356.

Therefore, the relationship between therapeutic alliance and symptom outcome was not moderated by IG-M. No link between alliance and symptomatology emerged, differing from previous studies (Karver et al., 2018). This may be due to our small sample (90 patients vs. the 108 required by the a priori power analysis; blinded). Furthermore, larger alliance-outcome effects have been found for externalizing vs. internalizing problems (Karver et al., 2018; Roest et al., 2023), and over 75% of our sample had an internalizing disorder. Additionally, high alliance scores may have caused ceiling effects, reducing variance and underestimating the alliance-symptom relationship (Mander et al., 2019; Meier, 2022). Of note, the present study used robust statistical methods (by acknowledging the hierarchical data structure) and assessed therapeutic alliance up to 24 times over the course of therapy, in contrast to many other studies that investigated therapeutic alliance only to a single time point (Roest et al., 2023). Meta-analytic results indicated that the association between change in child-therapist alliance and child outcome is weaker in studies that used more robust analytic strategies and in studies that measured alliance in each session, as in the present study (Roest et al., 2023). There is also an ongoing discussion about the appropriateness of using the therapeutic alliance as a predictor for treatment outcome, as outcome improvements in an early stage of therapy might lead to a stronger therapeutic alliance, which in turn might result in an overestimation of the alliance’s importance for treatment outcome (Roest et al., 2023). Nevertheless, it is beyond question that therapeutic alliance plays a central role within the psychotherapeutic progress (Roest et al., 2023; Cuijpers et al., 2019). More research is needed to disentangle the alliance-outcome relationship appropriately, especially with regard to the therapeutic work with children and adolescents.
